# Supplementary material for: Infancy‐onset diabetes caused by de‐regulated AMPylation of the human endoplasmic reticulum chaperone BiP
Source: EMBO Mol Med. 2023 Jan 27;15(3):e16491. doi: 10.15252/emmm.202216491 (PMC9994480; doi:10.15252/emmm.202216491)
Supplement: Supplementary file 1 — Expanded View Figures PDF [file EMMM-15-e16491-s003.pdf]

Expanded View Figures

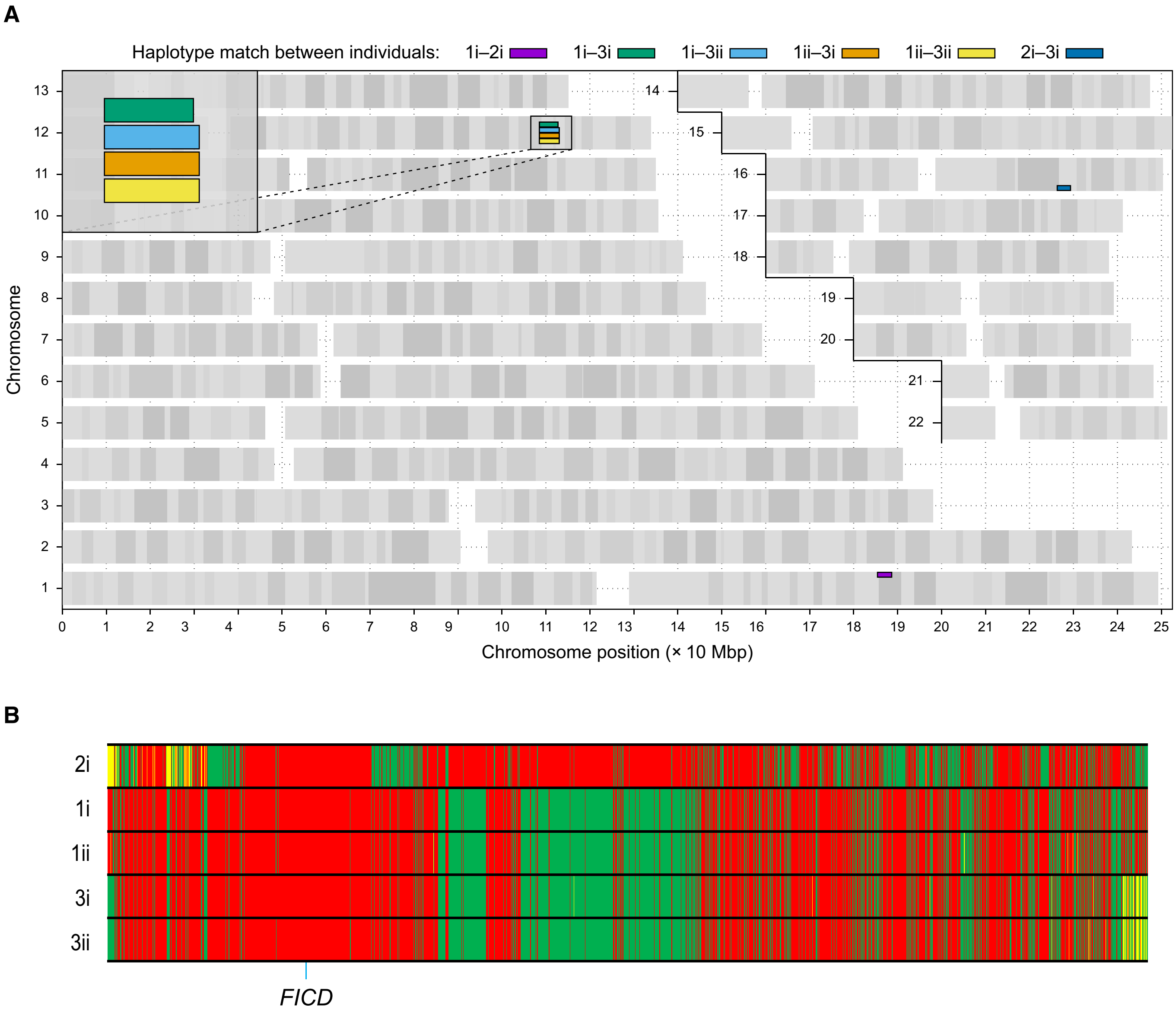

Figure EV1. Analysis of shared haplotypes amongst affected individuals homozygous for the p.(Arg371Ser) mutation in *FICD*.

A Graphical representation of genome-wide shared genomic segments larger than 3 Mbp in the five individuals with *FICD* homozygous mutations. Each colour bar represents a haplotype shared by two individuals (across all four copies of the same chromosome), labelled according to Fig 1A. The four rectangles on the long arm of chromosome 12 (inset) show that the four individuals in Families 1 and 3 share a haplotype [approximate coordinates chr12(hg19):108512514–113023258] including the *FICD* gene [chr12(hg19):108909051–108913380], but none of them shares a haplotype with the patient in Family 2 (individual 2i) —hence the lack of annotated purple or dark blue bars. Note, genotype information was not available for family member 2ii.

B Genotype calls for 2,735 single nucleotide, not multiallelic variants, located on chr12:(hg19):108500000–113100000 in the five individuals with the *FICD* homozygous p.(Arg371Ser) mutation. Only variants where at least one of the five patients carries the alternative allele are shown. Variants with a coverage of < 15 reads and an allele balance for heterozygous calls < 0.25 were removed. The position of the three variants in the *FICD* locus (including the pathogenic p.(Arg371Ser) variant) is indicated at the bottom of the graph. Green = homozygous for the reference allele, Yellow = heterozygous, Red = homozygous for the alternative allele. Note the similarity in variant pattern in affected individuals from families 1 & 3 and the dissimilarity with the affected member of family 2.

© 2023 The Authors

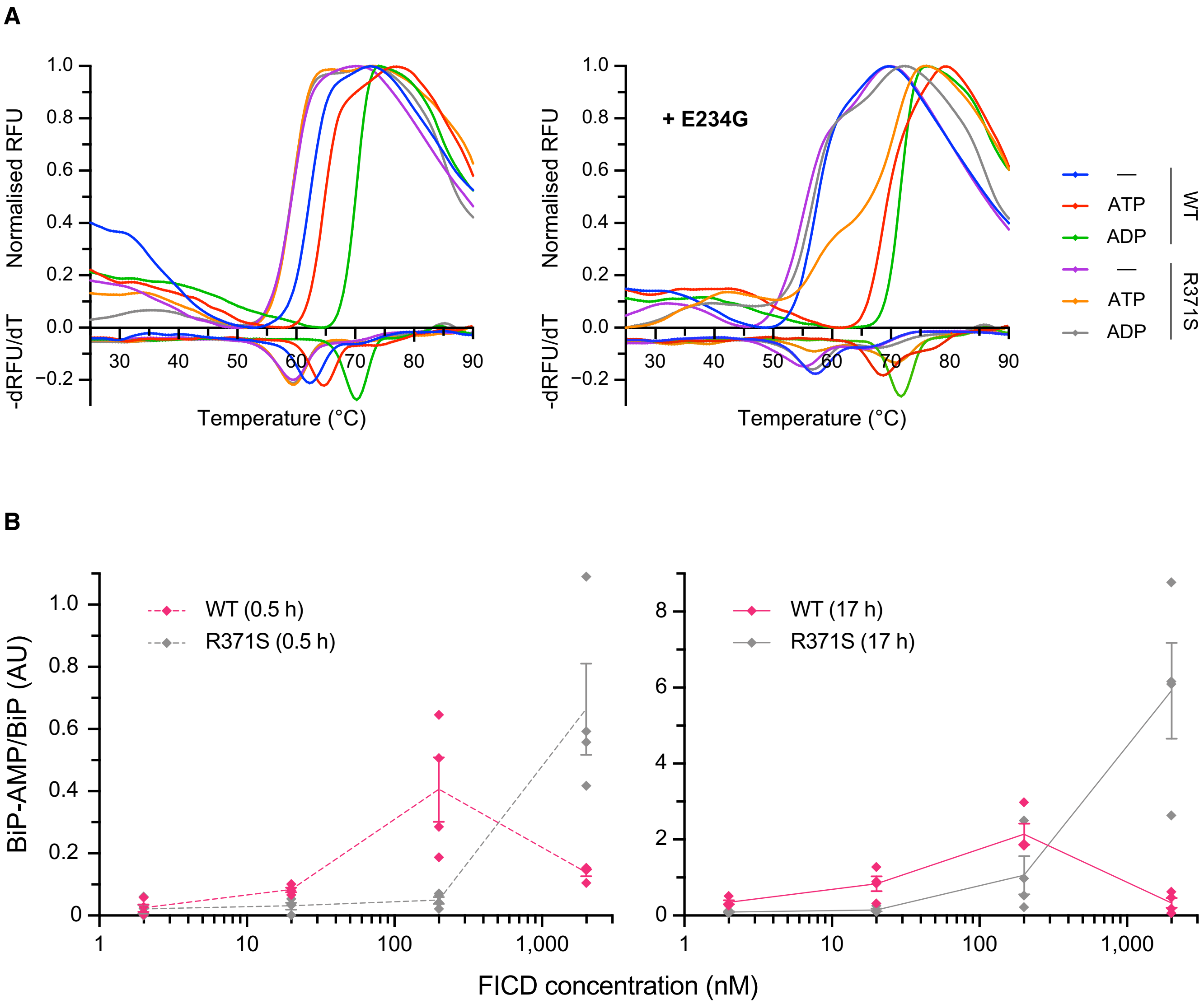

Figure EV2. Effect of the R371S mutation on stability and activity of FICD.

A Representative (of experiments reproduced three time), normalised differential scanning fluorimetry (DSF) melt curves of the indicated FICD proteins (1 μM) in presence and absence of nucleotide (2.5 mM), shown above their corresponding negative first-derivatives. Note, $FICD^{E234G\text{-}R371S}$ displays a non-uniform T_m shift in response to ATP.

B Quantification of the AMPylated BiP signals relative to total BiP of experiments displaying the same data in Fig 3C (mean values ± SEM, n = 4, biological replicates), but split into the two time points of the experiment and presenting the values from all four replicates.

 © 2023 The Authors
