# Supplementary material for: Infancy‐onset diabetes caused by de‐regulated AMPylation of the human endoplasmic reticulum chaperone BiP
Source: EMBO Mol Med. 2023 Jan 27;15(3):e16491. doi: 10.15252/emmm.202216491 (PMC9994480; doi:10.15252/emmm.202216491)
Supplement: Supplementary file 5 — PDF+ [file EMMM-15-e16491-s002.pdf]

Article

TRANSPARENT PROCESS OPEN ACCESS

EMBO Molecular Medicine

Infancy-onset diabetes caused by de-regulated AMPylation of the human endoplasmic reticulum chaperone BiP

Luke A Perera[1,†,‡], Andrew T Hattersley[2,†], Heather P Harding[1,†], Matthew N Wakeling[2,†], Sarah E Flanagan[2], Ibrahim Mohsina[3], Jamal Raza[3], Alice Gardham[4], David Ron[1,*,†] & Elisa De Franco[2,*,†]

Abstract

Dysfunction of the endoplasmic reticulum (ER) in insulin-producing beta cells results in cell loss and diabetes mellitus. Here we report on five individuals from three different consanguineous families with infancy-onset diabetes mellitus and severe neurodevelopmental delay caused by a homozygous p.(Arg371Ser) mutation in *FICD*. The *FICD* gene encodes a bifunctional Fic domain-containing enzyme that regulates the ER Hsp70 chaperone, BiP, via catalysis of two antagonistic reactions: inhibitory AMPylation and stimulatory deAMPylation of BiP. Arg371 is a conserved residue in the Fic domain active site. The FICD^{R371S} mutation partially compromises BiP AMPylation *in vitro* but eliminates all detectable deAMPylation activity. Overexpression of FICD^{R371S} or knock-in of the mutation at the *FICD* locus of stressed CHO cells results in inappropriately elevated levels of AMPylated BiP and compromised secretion. These findings, guided by human genetics, highlight the destructive consequences of de-regulated BiP AMPylation and raise the prospect of tuning FICD's antagonistic activities towards therapeutic ends.

Keywords diabetes mellitus; endoplasmic reticulum chaperone; mutation; nucleotidyltransferases; post-translational

Subject Categories Genetics, Gene Therapy & Genetic Disease; Metabolism; Neuroscience

DOI 10.15252/emmm.202216491 | Received 24 June 2022 | Revised 8 December 2022 | Accepted 9 December 2022 | Published online 27 January 2023

EMBO Mol Med (2023) 15: e16491

Introduction

Translocation channels, chaperones, protein modifying enzymes and membrane trafficking components all maintain protein folding homeostasis in the endoplasmic reticulum and contribute to the integrity of secretion in eukaryotes (reviewed in: Braakman & Hebert, 2013). Whilst components of this machinery are common to most cells, its dysfunction in animals often manifests prominently as failure of the insulin-producing pancreatic beta cells to keep up with the needs of the organism. Although the basis for this apparent hypersensitivity of beta cells to ER stress is incompletely understood, rare mutations in 10 genes encoding components of this general ER proteostasis network are known to cause monogenic forms of diabetes (reviewed in: Shrestha *et al*, 2021; Yong *et al*, 2021). Given the broad role of protein secretion in animal physiology, these monogenic diseases are enriched in extra-pancreatic manifestations, often resulting in syndromic forms of diabetes mellitus associated with developmental abnormalities (reviewed in: Sanchez Caballero *et al*, 2021).

The Hsp70 chaperone BiP is an essential cog of the ER proteostasis network (reviewed in: Pobre *et al*, 2019). BiP is encoded by an essential gene, *HSPA5*, whose inactivation leads to early embryonic lethality in mice (Luo *et al*, 2006) but loss-of-function alleles in several BiP co-factors are associated with beta cell dysfunction (Synofzik *et al*, 2014; Danilova *et al*, 2019). Thus, beta cells are sensitive to disruption of BiP's chaperone network.

Unique amongst Hsp70 chaperones, BiP is regulated by AMPylation, a reversible covalent modification by which an AMP moiety from ATP is transferred onto a protein's hydroxylated side chain. AMPylation (also known as adenylylation) is extensively exploited by bacteria to regulate endogenous processes (Kingdon *et al*, 1967) or host proteins in the context of infection (reviewed in: Woolery

1 Cambridge Institute for Medical Research, University of Cambridge, Cambridge, UK
2 Institute of Biomedical and Clinical Science, College of Medicine and Health, University of Exeter, Exeter, UK
3 Department of Endocrine and Diabetes, National Institute of Child Health, Karachi, Pakistan
4 North West Thames Regional Genetics Service, Harrow, UK
*Corresponding author. Tel: +44 01223 768 940; E-mail: dr360@medschl.cam.ac.uk
**Corresponding author. Tel: +44 01392 408 232; E-mail: e.de-franco@exeter.ac.uk
†These authors contributed equally to this work
‡Present address: The Francis Crick Institute, London, UK

© 2023 The Authors. Published under the terms of the CC BY 4.0 license.

et al, 2010). Animal genomes encode a single ortholog of the widely disseminated family of Fic domain proteins that carry out AMPylation in bacteria (Worby *et al*, 2009; Yarbrough *et al*, 2009). Animal FICD is an ER-localised type II transmembrane protein whose active site faces the lumen. There, it single-handedly carries out two functionally antagonistic reactions: BiP AMPylation (Ham *et al*, 2014; Sanyal *et al*, 2015; Preissler *et al*, 2015b) and deAMPylation (Casey *et al*, 2017; Preissler *et al*, 2017a).

AMPylation, which occurs on Thr518 (Preissler *et al*, 2015b), locks BiP in a conformation with low substrate affinity and impaired responsiveness to the stimulatory effect of J-domain co-factors, effectively neutralising the chaperone (Preissler *et al*, 2015b, 2017b). This biochemical feature is consistent with earlier observations that levels of modified (now known to be AMPylated) BiP are regulated physiologically: decreasing with mounting ER stress and increasing with stress resolution (fig 1 in Hendershot *et al*, 1988; figs 1 and 3 in Laitusis *et al*, 1999). This balancing act is critically dependent on FICD (Casey *et al*, 2017; Preissler *et al*, 2017a) and arises from fine tuning of the enzyme's active site to reciprocally regulate its two antagonistic functions (Perera *et al*, 2019, 2021).

Despite advances in the biochemical analysis of BiP AMPylation, its consequences in terms of organismal physiology are less well understood. Photosensitivity has been reported in flies lacking *ficd* (Rahman *et al*, 2012; Moehlman *et al*, 2018), but in knockout mice the phenotype is limited to weak immunological and learning deficits (McCaul *et al*, 2021) or mild hypersensitivity to experimental stress conditions (Casey *et al*, 2022). Here, we identify a rare missense, recessive mutation within FICD's active site in five individuals diagnosed with infancy-onset diabetes mellitus and neurodevelopmental abnormalities. This finding provides insight into the functional consequences of deregulated BiP AMPylation.

Results

The homozygous *FICD* p.(Arg371Ser) mutation lead to infancy-onset diabetes with neurodevelopmental abnormalities

Two siblings, born to related parents, were referred to the Exeter genomics laboratory for genetic testing on suspicion of Wolcott Rallison syndrome, a disorder caused by recessive variants in the *EIF2AK3* gene that encodes a regulator of ER proteostasis, PERK (Delepine *et al*, 2000). The proband (Fig 1A) was diagnosed with diabetes at the age of 23 weeks. They also had severe developmental delay and skeletal abnormalities were suspected when they were 5 years old. Their older sibling was similarly affected and died aged 7 (cause unknown). Analysis of known neonatal diabetes genes, including *EIF2AK3*, did not identify a likely causative variant. In search of novel genetic causes of monogenic diabetes, whole genome sequencing analysis of the two siblings was carried out. The two shared 13 rare homozygous or hemizygous coding variants (Table EV1). None of these variants were located within genes

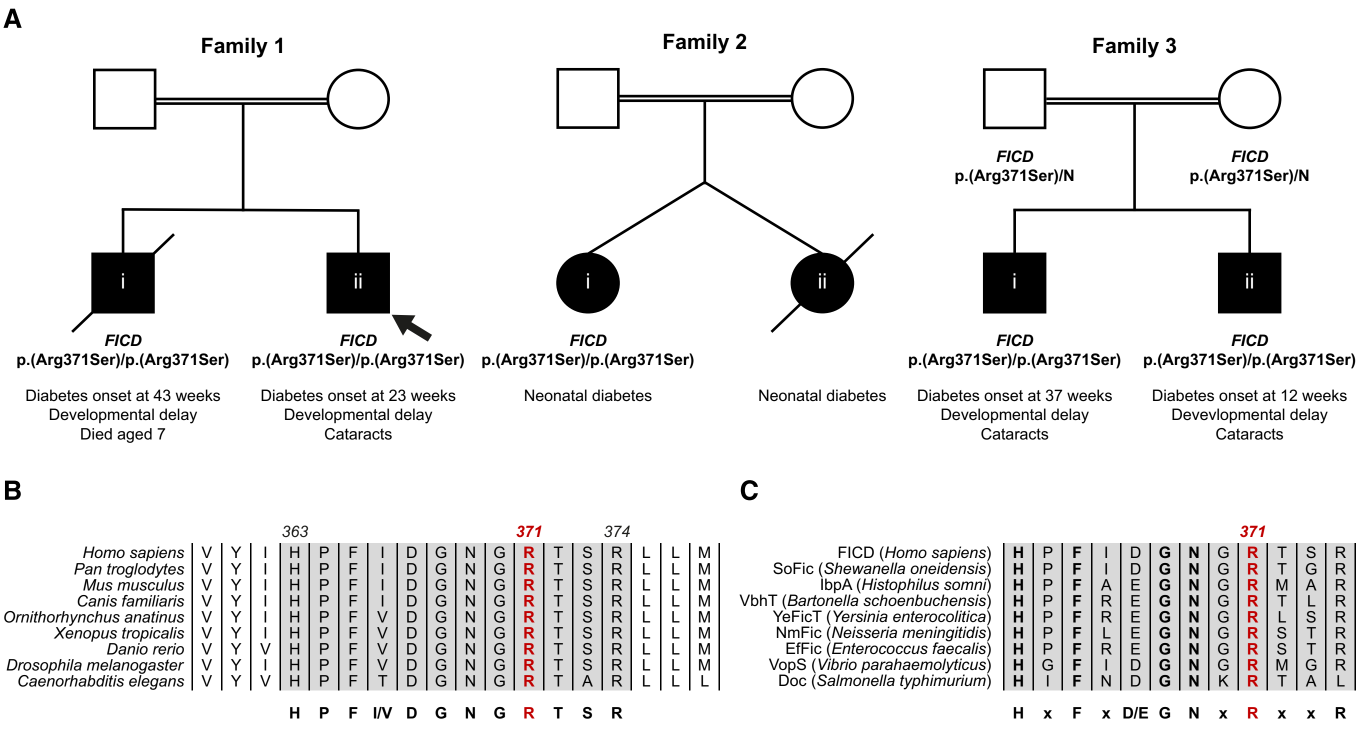

Figure 1. A homozygous p.(Arg371Ser) mutation within the human *FICD* gene results in infant-onset diabetes and severe neurodevelopmental delay.

A Partial pedigrees of three families found to bear this rare genetic variant. Where available, information pertaining to the *FICD* genotype and the phenotype of affected individuals are shown. The first individual identified to carry this homozygous mutation (the proband, family member 1ii) is indicated with an arrow.

B, C Arg371 is located within the catalytic Fic motif and is highly conserved both across metazoan FICD orthologues (B) and across the wider Fic and Doc protein superfamily (C). The Fic motif is highlighted in grey, and residue numbering corresponds to human FICD.

 © 2023 The Authors

Table 1. Summary of clinical features of the five individuals homozygous for the *FICD* p.(Arg371Ser) variant. The number of individuals for whom information on each feature was available is indicated (*n*).

Cohort feature	
Sex (n = 5)	Male (4)
Age at last assessment (mean, years)	5.2 (range 0–9)
Birth weight (mean, g) (n = 3)	3,063 (range 2,800–3,460)
Gestation (mean, weeks) (n = 3)	40 (range 38–41)
Infancy-onset diabetes (n = 5)	5
Age at diabetes onset (mean, weeks) (n = 4)	29 (range 12–43)
Insulin dose (mean, U/kg/day) at last assessment (n = 3)	1.0 (range 0.7–1.2)
Developmental delay (n = 4)	4
Cataracts (n = 4)	3
Short stature (n = 4)	2

previously linked to diabetes. Next, we investigated whether any of these 13 genes had been previously found to harbour homozygous or hemizygous variants in a cohort of 126 individuals diagnosed with diabetes before the age of 12 months, whose genome had been sequenced but no causative mutation discovered. This analysis revealed that one of the variants identified in the two siblings, the NM_007076.2:c.1113G>C, p.(Arg371Ser) in the *FICD* gene, had been previously detected in three additional individuals from two families, taking the total number of individuals homozygous for this variant to 5, all diagnosed with neonatal/infancy-onset diabetes (Fig 1A and Table 1).

The p.(Arg371Ser) variant is very rare [minor allele frequency = 3.98×10^{-6} in the GnomADv2 database (Karczewski *et al*, 2020)] and it was not found in the homozygous state in either the '100,000 genomes', 'Gene and health', 'GnomAD', 'BioBank', or 'Decipher' data sets. The p.(Arg371Ser) variant affects a residue located within the catalytic Fic domain motif and, as such, it is highly conserved both across metazoan FICD protein homologues (Fig 1B) and across the wider Fic and Doc (Fido) protein superfamily (Fig 1C).

Since the five individuals were homozygous for the same *FICD* variant, we investigated whether they shared a haplotype consistent with them being related and having inherited the variant from a common ancestor. Analysis of the whole genome sequencing data revealed that the four affected individuals in Family 1 and Family 3 shared a 4.5 Mb haplotype that includes the *FICD* locus, suggesting that their *FICD* p.(Arg371Ser) variant was inherited from a common distant ancestor (Fig EV1). Within the haplotype, they shared three rare variants, of which *FICD* p.(Arg371Ser) was the only one predicted to affect the encoded protein. Importantly, the individual in Family 2 did not share a haplotype with the other two families, suggesting that the *FICD* variant is likely to have arisen independently in that family.

The clinical features of the five individuals homozygous for the *FICD* p.(Arg371Ser) mutation are summarised in Table 1. All were diagnosed with diabetes before the age of 12 months and needed replacement insulin therapy. The proband in Family 2 had a sibling who was also diagnosed with diabetes but died in the neonatal period (no DNA was available). The birth weight in this cohort was within the normal range (mean 3,063 g, range 2,800–3,460 g), consistent with these patients not having a severe reduction in foetal insulin secretion in utero. None of the parents in the three families (who are obligate heterozygous carriers of the *FICD* mutation) had diabetes.

Severe developmental delay was diagnosed in all the affected children except for one (the proband in Family 2 who could not be re-assessed after insulin-dependent diabetes was discovered in infancy). 3 of the 5 affected children developed bilateral cataracts. Additional features such as short stature, skeletal abnormalities, microcephaly and deafness were also observed but were not seen in more than one family; therefore, it is unclear whether they are features of the FICD-related disease.

The identification of the same rare variant in three families, across two separate haplotypes, with similar clinical features, supports the *FICD* p.(Arg371Ser) mutation being responsible for the observed phenotype.

The Arg371Ser mutation compromises both enzymatic activities of FICD *in vitro* but leads to dominance of BiP AMPylation over deAMPylation

Arg371 is an active site residue conserved in FICD and in the very large family of bacterial Fic-domain proteins and the wider Fido superfamily (Fig 1B and C) (Kinch *et al*, 2009; Khater & Mohanty, 2015). In AMPylating FICD, Arg371 contributes to the binding of the β-phosphate of ATP, a co-substrate in the reaction, and presumably in AMPylation transition-state stabilisation (Fig 2A). In the deAMPylating enzyme, Arg371 engages the side chain of Glu234 in the conformation necessary for Glu234 to position a catalytic water molecule in-line for nucleophilic attack into the phosphodiester bond linking BiP(Thr518) and the AMP moiety (Fig 2B). Thus, the Arg371Ser mutation is poised to compromise both reactions.

The antagonistic nature of the two reactions implies that the phenotype arising from the mutation is likely to depend on the extent to which each is compromised. To address this issue, we expressed the structured luminal domains of wild-type and Arg371Ser mutant FICD in *E. coli* and evaluated the purified enzymes *in vitro*. Nucleotide-free $FICD^{R371S}$ possesses a modestly lower melting temperature than the wild-type enzyme, consistent with the stabilising effect of an ionic interaction between the side chains of Arg371 and Asp367 noted in the crystal structure of the apo wild-type enzyme (PDB 4U04). Importantly, whereas the thermal stability of the wild-type enzyme increased significantly upon binding ATP and even more so ADP, the stability of the $FICD^{R371S}$ mutant remained unchanged (Figs 3A and EV2). This finding is consistent with an important role for the Arg371 sidechain in contacting the β-phosphate of ADP or ATP bound in an AMPylation competent conformation or the γ-phosphate of ATP bound in an AMPylation incompetent conformation (Fig 2A; Perera *et al*, 2019). Thus, the Arg371Ser mutation is likely to both lower the affinity of the FICD for nucleotide and reduce the protein-stabilising effect of any bound nucleotide di- or tri-phosphate. The $FICD^{R371S}$ mutant enzyme nonetheless retains some ability to bind nucleotide, as evidenced by the marked protein-stabilising effect of exogenous ATP when the Fic

© 2023 The Authors

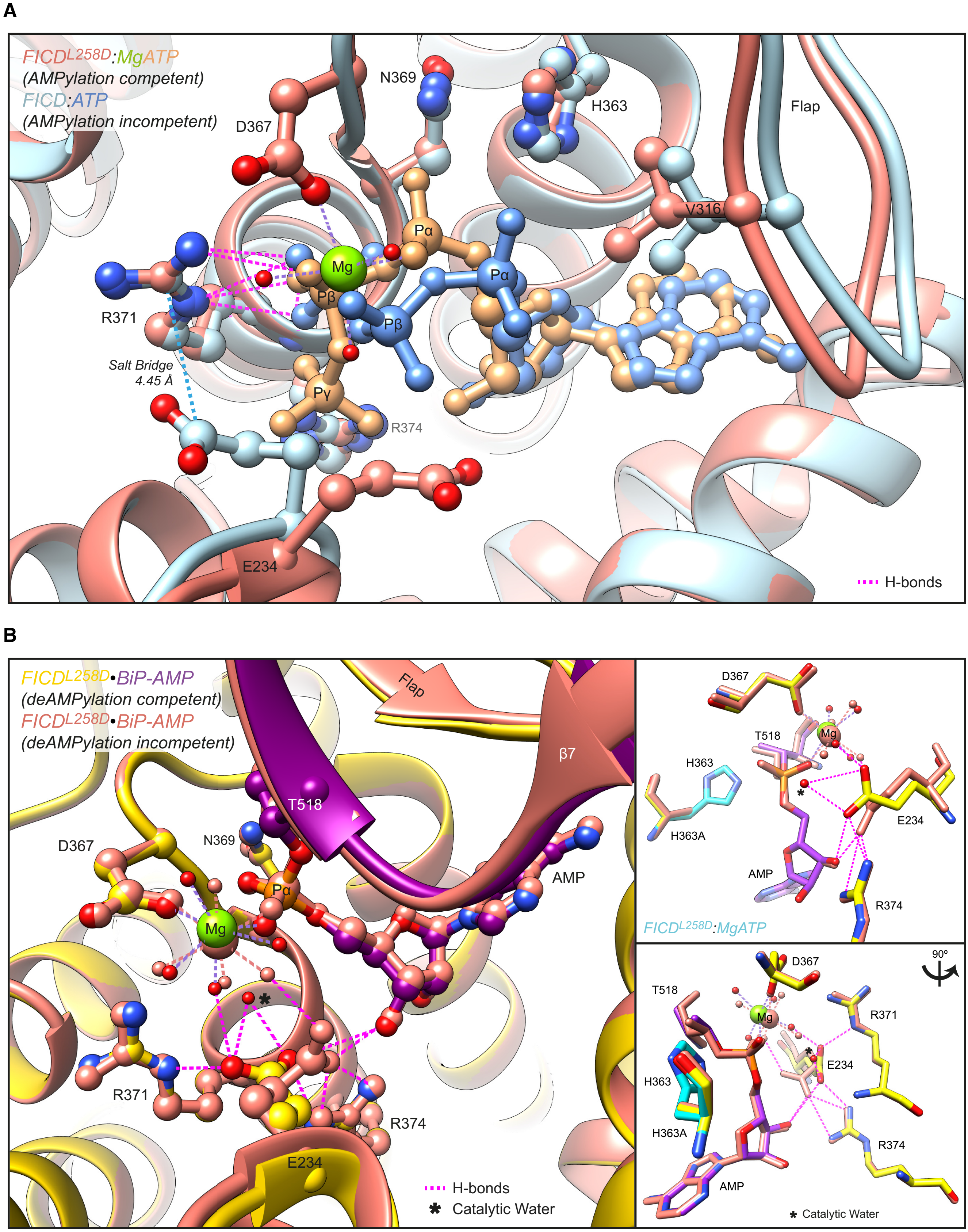

Figure 2.

 © 2023 The Authors

Figure 2. Mutation of Arg371 is predicted to perturb both FICD-catalysed AMPylation and deAMPylation.

A Structures of monomeric (FICD^{L258D}) and dimeric FICD proteins, crystallised in the presence of a large excess of magnesium cations and ATP, are superposed (PDB 6I7K and 6I7G, respectively). The former engages MgATP in an AMPylation competent conformation and the latter engages (only) ATP in an AMPylation incompetent conformation (Perera *et al*, 2019). Arg371 forms hydrogen bonds with both nucleotides (pink dashed lines). Arg371's interaction with the β-phosphate of MgATP is also likely to help stabilise the AMPylation transition state. A potential long range ionic interaction between Arg371 and Glu234 in the non-competent conformation is also annotated.

B Two alternative states of FICD's active site engaged with its deAMPylation substrate, BiP-AMP. Glu234 can exist in at least two conformations. In the deAMPylation competent conformation, Glu234 correctly orientates a catalytic water molecule (*) for in-line nucleophilic attack into the backside of the BiP-AMP phosphodiester bond (PDB 7B7Z). In the deAMPylation incompetent conformation Glu234 points away from the position of this putative catalytic water (PDB 7B80) (Perera *et al*, 2021). Hydrogen bonds formed by Glu234 are annotated in both cases (pink dashed lines). Arg371 interacts with Glu234 only when it resides in a deAMPylation competent conformation. Insets, reduced view of the superposed active sites, shown in orthogonal views, additionally overlaid with the catalytic His363 residues from PDB 6I7K (cyan).

domain gatekeeper glutamate residue, that normally limits ATP affinity and inhibits AMPylation (Engel *et al*, 2012; Bunney *et al*, 2014), is also mutated (FICD$^{E234G\text{-}R371S}$; Figs 3A and EV2). Together these finding point to an important role of the Arg371Ser mutation in modifying the active site and leaves open the possibility of some residual enzymatic activity.

It was previously found that the oligomeric state of wild-type FICD reciprocally regulates its functionally opposed enzymatic activities (Perera *et al*, 2019). However, at the concentrations tested, both wild-type and mutant FICD appear principally dimeric (as assessed by size exclusion chromatography, Fig 3B), consistent with previous findings pertaining to wild-type FICD (Bunney *et al*, 2014; Perera *et al*, 2019). This suggests that differences in oligomeric state are unlikely to account for disparities between their relative enzymatic activities.

The antagonistic activities of wild-type FICD are subordinate to its oligomeric state: the dimer is a better deAMPylase and the monomer a better AMPylase (Perera *et al*, 2019). Given a K_d of FICD dimerisation in the nanomolar range, the relationship between the amount of AMPylated BiP and the FICD enzyme concentration, produced by reactions not otherwise limited by ATP, is biphasic—FICD's AMPylation activity dominates at low (nanomolar) enzyme concentrations whilst its deAMPylation activity dominates at higher enzyme concentrations (Perera *et al*, 2019). This biphasic relationship is lost in reactions catalysed with FICD^{R371S}. Moreover, at both early and late time points, more AMPylated BiP is recovered in reactions performed with the wild-type enzyme at low FICD concentrations, but this difference is reversed at higher FICD concentrations (Figs 3C and D and EV2B).

This situation is consistent with a defect in AMPylation activity of the mutant enzyme, which is perhaps most clearly demonstrated by the difference in BiP-AMP accumulation catalysed by low concentrations of FICD at early time points (conditions which are likely to report on the initial AMPylation rate of each enzyme). Nevertheless, the greater accumulation of AMPylated BiP catalysed by higher concentrations of FICD^{R371S}, relative to FICD^{WT}, is indicative of an even more significant impact of the mutation on FICD-mediated deAMPylation. Indeed, comparing the rate of BiP deAMPylation under substrate saturating conditions, by the wild-type and FICD^{R371S} enzyme, reveals robust activity of the former and no measurable deAMPylation activity of the latter (Fig 3E).

The absence of measurable deAMPylation activity coupled with weak AMPylation activity of the FICD^{R371S} enzyme explains the accumulation of BiP-AMP over time. These features are also consistent with the observation that even sub-stoichiometric concentrations of wild-type FICD can limit the accumulation of AMPylated BiP in reactions that contain the mutant enzyme (Fig 3F). The implications of these *in vitro* observations to the mode of inheritance of the functional deficits are discussed below.

FICD^{R371S} de-regulates BiP-AMP levels and compromises secretion

Cells lacking FICD are unable to deAMPylate BiP (Casey *et al*, 2017; Preissler *et al*, 2017a). Therefore, the *in vitro* experiments above suggest that in cells expressing FICD^{R371S} as their only source of BiP-modifying enzymatic activity, the deAMPylation-unopposed, weak AMPylation function of the mutant FICD might induce a hyperAMPylated pool of BiP. To test this idea, we transiently introduced wild-type and Arg371Ser mutant FICD into CHO cells lacking endogenous FICD and measured the effects on BiP AMPylation, using a recently described monoclonal antibody reactive with AMPylated proteins (MoAb 17G6, Hopfner *et al*, 2020).

Expression of wild-type FICD led to no detectable BiP-AMP signal, reflecting the dominance of its deAMPylating activity (Preissler *et al*, 2017a). By contrast, the Arg371Ser mutant FICD promoted a conspicuous pool of AMPylated BiP (Fig 4A, compare lanes 2 & 4). The AMPylating activity of the Arg371Ser mutant was dependent on the integrity of its active site, as no BiP-AMP signal was detectable in cells expressing the double Arg371Ser-His363Ala mutant. As expected, levels of AMPylated BiP were even higher in cells expressing the deregulated Glu234Gly mutant FICD, which possesses both enhanced AMPylating activity and no detectable deAMPylating activity (Preissler *et al*, 2017a). The promiscuous features of FICD^{E234G} likely account for its auto-AMPylation and lower expression compared to the other FICD variants (Fig 4A, lane 3). The FICD immunoblot also reveals a minor, lower-mobility species in cells expressing FICD^{R371S} (Fig 4A, compare lane 2 with lanes 4 & 5, marked by an asterisk) likely reflecting cryptic glycosylation of Asn369, exposed by the mutation (see Discussion). Only the FICD^{E234G} cells manifested significant activation of their CHOP::GFP and XBP1::Turquoise unfolded protein response (UPR) reporters (Fig 4B and C), an observation consistent with a requirement for high levels of AMPylation to yield basal activation of the UPR (Perera *et al*, 2019).

To explore the consequences of more physiological expression levels of FICD^{R371S}, we used CRISPR/Cas9 mediated homologous recombination to replace the endogenous wild-type FICD of CHO cells with an Arg371Ser mutant (Fig 5A).

The monoclonal antibody, reactive with AMPylated proteins, revealed a strong signal of 75 kDa in immunoblot of lysate from

© 2023 The Authors

cycloheximide-treated wild-type CHO cells and weaker signal in untreated cells (Fig 5B, left panel) a pattern consistent with previous observations (Laitusis *et al*, 1999; Preissler *et al*, 2015b). The identity of the signal with AMPylated BiP was confirmed by performing the same assay on BiP immunopurified from cells with a BiP-specific antibody (Fig 5B, right panel). $FICD^{R371S}$ cells had a different BiP AMPylation profile, with a consistent baseline signal (compared with a variable, often undetectable signal in wild-type cells), that

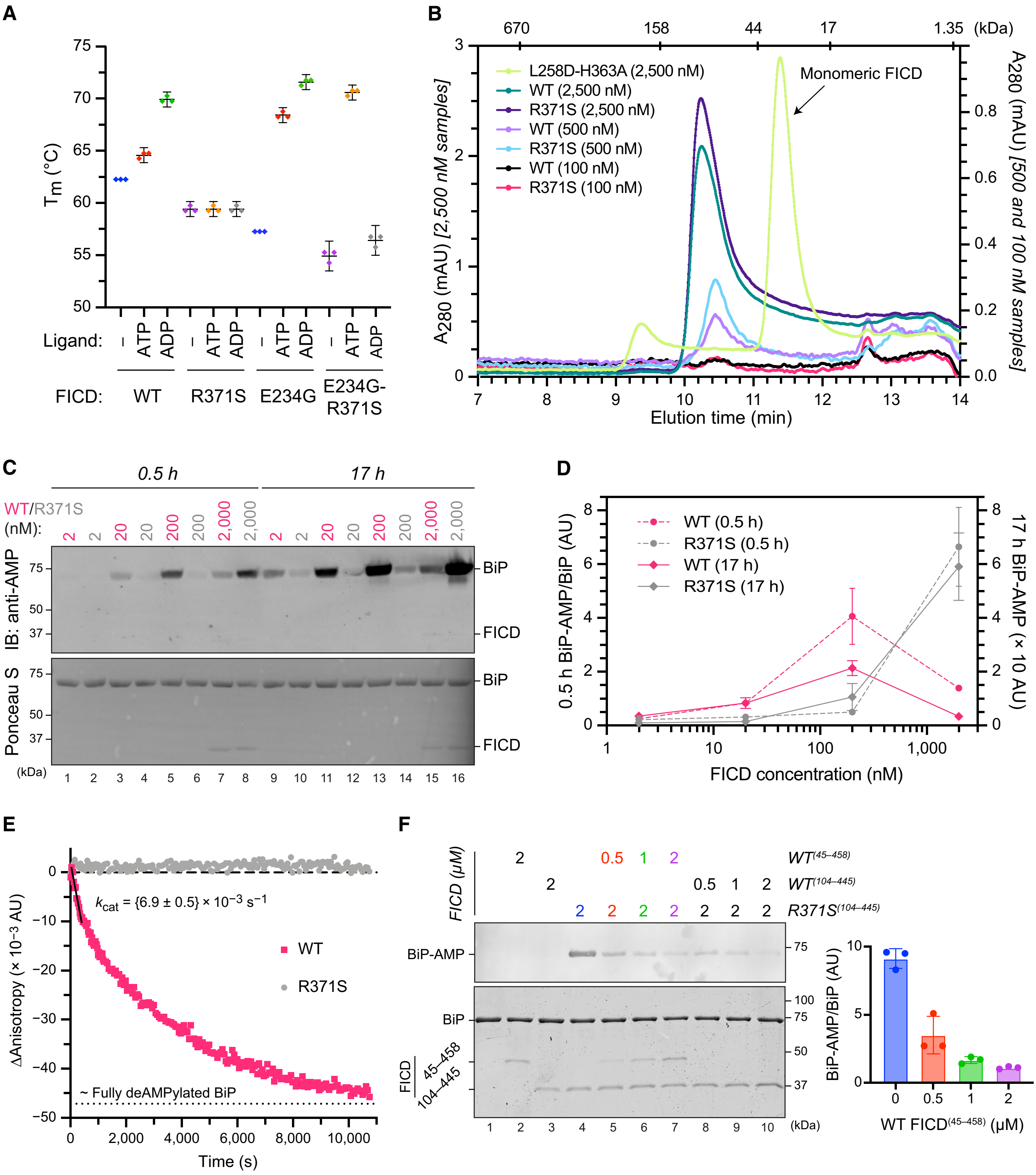

Figure 3.

 © 2023 The Authors

Figure 3. The FICD Arg371Ser mutation compromises BiP AMPylation and eliminates all detectable BiP deAMPylation *in vitro*.

A Plot of the principal melting temperatures (T_ms) of wild-type FICD (WT) and FICD^{R371S} in the presence or absence of nucleotide ligands and/or FICD's gatekeeper Glu234 residue, derived from differential scanning fluorimetry (DSF). The mean T_m ± 95% confidence interval (CI) is displayed, from n = 3 independent experiments. Arg371Ser mutation slightly destabilises the apo enzyme and significantly reduces the ability of ATP and ADP to stabilise FICD. Glu234Gly mutation partially restores ATP (but not ADP) stabilisation of FICD$^{E234G\text{-}R371S}$ (see Fig EV2A).

B Size exclusion chromatography confirms that the oligomerisation tendency of FICD^{R371S} is unperturbed relative to wild-type FICD. Across a range of assessable enzyme concentrations both FICDs elute as predominantly dimeric species. The elution profile of a monomeric FICD$^{L258D\text{-}H363A}$ mutant is also shown for reference, alongside the elution times of molecular weight standards.

C Representative immunoblot comparing the ability of wild-type and Arg371Ser FICD (at a wide range of enzyme concentrations) to catalyse the accumulation of BiP-AMP in the presence of physiological ATP concentrations (5 mM). At low enzyme concentrations (and early time points) the AMPylation defect imposed by Arg371Ser is manifest. At higher enzyme concentrations where the deAMPylation activity of the wild-type enzyme begins to dominate, the residual AMPylation activity of FICD^{R371S} results in a greater accumulation of BiP-AMP (relative to the same concentration of wild-type enzyme).

D Quantification of the AMPylated BiP signals, relative to total BiP, from experiments as in (C) (mean values ± SEM, n = 4, biological replicates). Figure EV2B displays the values from all four repetitions.

E A fluorescence polarisation-based deAMPylation assay highlighting the lack of activity catalysed by FICD^{R371S}. Enzyme-saturating concentrations of BiP-AMP were provided as deAMPylation substrate. No enzymatic activity of 10 μM (grey trace, shown) or 20 μM enzyme FICD^{R371S} was detectable. The estimated fluorescence anisotropy value of a fully deAMPylated BiP sample is derived from a heuristic fitting of a single exponential decay curve. The k_{cat} value for wild-type FICD was calculated from n = 3 biological replicates (mean ± SEM).

F Representative immunoblot comparing the accumulation of BiP-AMP in reactions constituted with the indicated concentrations of wild-type and Arg371Ser FICD, performed as in (C) (upper left panel). The lower left panel is a Coomassie-stained gel of the reaction constituents. An N-terminally extended version of the wild-type enzyme (residues 45–458 was used in lanes 2 and 5–7, to distinguish its migration on the gel from the mutant (residues 104–445). The bar diagram provides quantification of the AMPylated BiP signals relative to total BiP (from n = 3, biological replicates, mean values ± SEM).

increased only modestly upon cycloheximide treatment. Conversely, cells lacking FICD activity altogether (*FICD*$^{\Delta}$ cells) had only a background signal in the immunoblot, consistent with FICD's essential role in BiP AMPylation (Preissler *et al*, 2015b).

To explore further consequences of the *FICD*^{R371S} mutation on the regulation of BiP AMPylation, we exposed wild-type and mutant cells to thapsigargin, an agent that induces ER stress by luminal calcium depletion. In wild-type cells the imposition of ER stress markedly lowered the BiP-AMP signal (compare lanes 1 with 3 and 5 with 6, Fig 5C; as previously noted: Laitusis *et al*, 1999). By contrast, in *FICD*^{R371S} mutant cells the imposition of ER stress had a much weaker effect on BiP-AMP levels (compare lanes 7 with 9 and 11 with 12, Fig 5C and D). These observations are consistent with impaired ER stress-mediated de-AMPylation in the mutant cells and suggest the potential for defective proteostasis arising from their inability to adjust functional levels of BiP to fluctuating levels of ER stress.

Under certain experimental conditions FICD can AMPylate proteins other than BiP (Truttmann *et al*, 2016, 2017; Hopfner *et al*, 2020; McCaul *et al*, 2021). To further explore the role of BiP hyperAMPYlation in the functional consequences of deregulated FICD, we exploited recent insights into the mode of BiP engagement by FICD: Complementarity between the proximal surface containing the substrate residue BiP^{T518} and FICD's active site is insufficient for AMPylation or deAMPylation. BiP-specific enzymatic activity relies on the integrity of FICD's accessory TPR domain, which interacts specifically with domain-docked BiP. Point mutations in FICD's TPR domain (K124E, H131A) that compromise BiP binding have no effect on the enzyme's active site but abolish its ability to use BiP as a substrate (Fauser *et al*, 2021; Perera *et al*, 2021). Therefore, we compared the secretion of a model protein (secreted alkaline phosphatase, SEAP) upon co-expression of FICD enzymes which possess either wild-type or compromised TPR domains—mutations K124E and H131A that abolish BiP interaction. These experiments, carried out in *FICD*$^{\Delta}$ cells, reveal that compromised secretion by deregulated alleles of FICD is sensitive to mutations that weaken their interaction with BiP (Fig 6A–C). The existence of other substrates whose hyperAMPylation might contribute to the genetic disorder cannot be excluded. However, these finding indicate that compromised secretion—a pathomechanism plausibly operative in the patients—relies on features of FICD that endow it with selectivity towards BiP.

Discussion

The findings presented here establish a specific recessive missense mutation [*FICD* p.(Arg371Ser)] in a gene encoding an ER-localised, protein modifying enzyme, as causing a previously unrecognised genetic syndrome characterised by infancy-onset diabetes mellitus and neurodevelopmental defects. The disease mechanism likely consists of an intra-organellar perturbation that compromises both insulin-producing beta cells and cells relevant to neurological development and/or function. Patients with pathogenic mutations in other genes known to cause diabetes through dysregulation of ER

Figure 4. FICD^{R371S} promotes accumulation of AMPylated BiP in CHO cells lacking endogenous FICD.

A Immunoblots of AMPylated proteins detected with a pan-AMP antibody, BiP, FICD and eIF2α (a loading control) in lysates of *FICD*$^{\Delta}$ CHO cells transiently transfected with plasmids encoding the indicated derivatives of FICD. Signals corresponding to AMPylated BiP, auto-AMPylated FICD^{E234G}, total BiP, FICD and eIF2α are indicated. The novel species reactive with the anti-FICD antiserum in lysates of cells transfected with plasmids expressing FICD^{R371S} (*) likely reflects a glycosylated isoform arising from the creation of a new glycosylation site at Asn369.

B Histograms of Integrated Stress Response reporter CHOP::GFP and XBP::Turquoise (XBP::Turq, a reporter of the IRE1 branch of the UPR) fluorescence in *FICD*$^{\Delta}$ CHO dual reporter cells transfected with expression vectors as in (A). Transfected cells were gated for mCherry positivity, a marker carried on the expression plasmid in *trans* to the indicated FICD effector.

C Quantification of the data from repeats of the experiment as shown in (B). The upper two graphs show the percent of cells with high CHOP::GFP or high XBP::Turq for each transfection. The lower panel provides quantitation of the median fluorescence intensity for each reporter. Mean ± SD, n = 4, biological replicates.

© 2023 The Authors

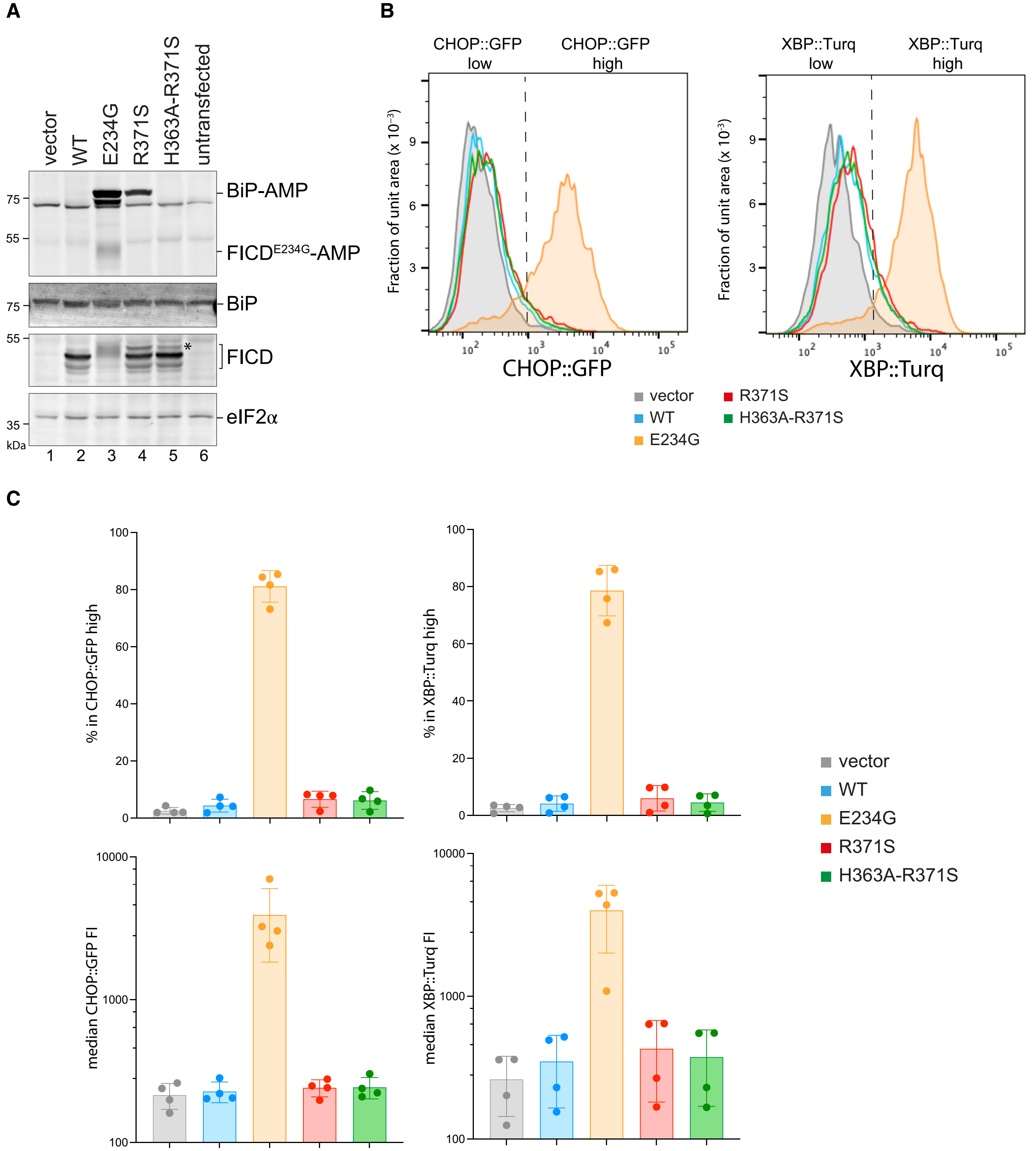

Figure 4.

function also have early-onset insulin-requiring diabetes and neurodevelopmental features (Shrestha *et al*, 2021). The identification of the same mutation in three families suggests a mutation-specific mechanism, as opposed to overall loss of gene function. A biochemical analysis of the effects of the Arg371Ser mutation on FICD's enzymology and the extant understanding of the ER chaperone BiP, a natural FICD substrate, suggest a mechanism for the underlying molecular pathology.

© 2023 The Authors

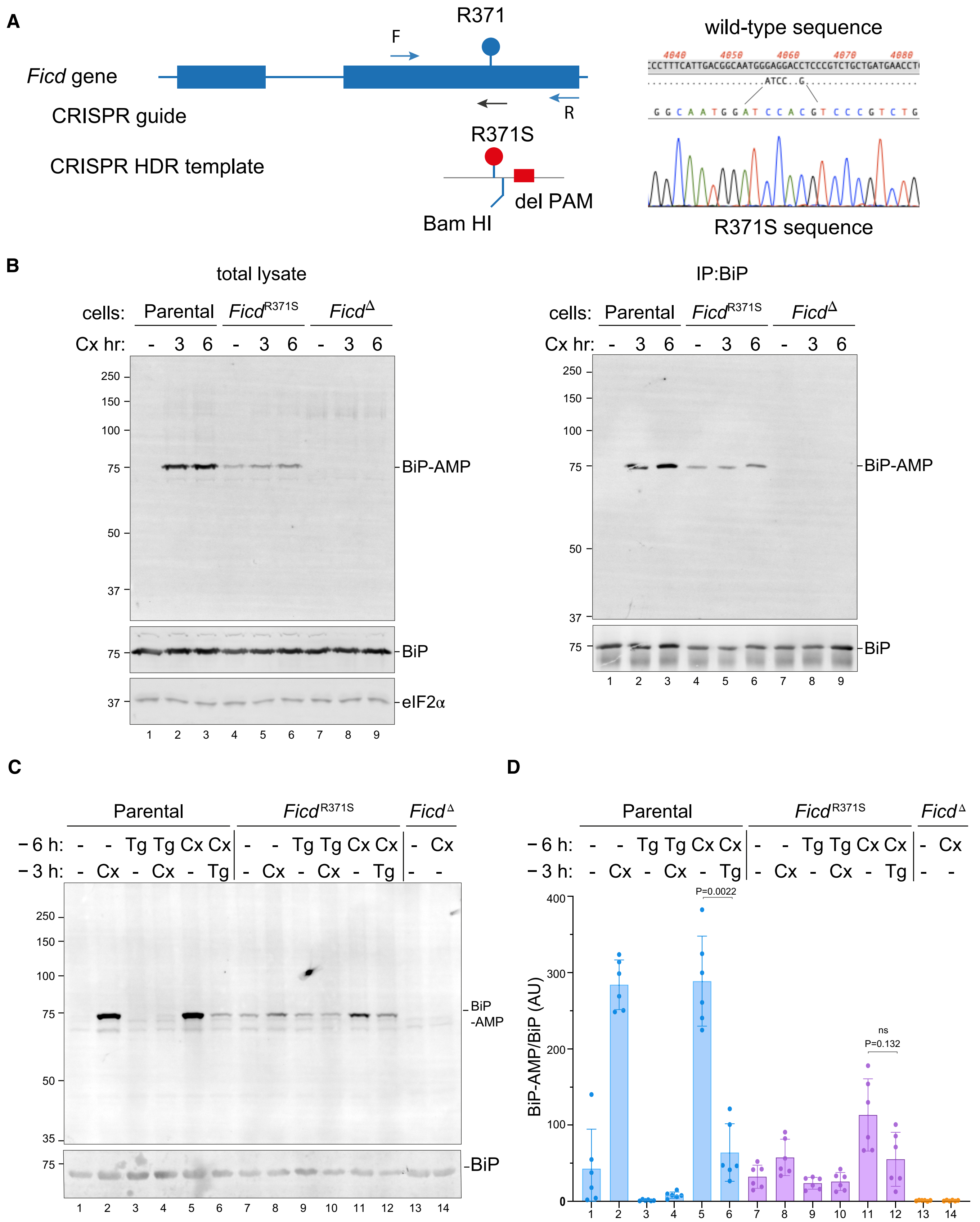

Figure 5.

© 2023 The Authors

◀ **Figure 5. Expressed at the endogenous *FICD* locus, the p.(Arg371Ser) mutation leads to defective clearance of AMPylated BiP in ER stressed cells.**

A On the left is a schema of the Chinese Hamster Ovary cell *FICD* locus. Exons depicted as blue boxes, introns as lines. Forward (F) and reverse (R) primers used to genotype the locus and the guide RNA that directs the Cas9 nuclease (CRISPR guide) are depicted by arrows. A schema of the repair template is provided below, with the Arg371Ser mutation, the silent *Bam*HI site and silent PAM-destroying features noted. To the right is the sequence of the locus before and after recombination and a sequencing trace of the locus in the mutant $FICD^{R371S}$ derivative cell line.

B On the left are immunoblots of AMPylated proteins detected with pan-AMP, BiP and eIF2α (a loading control) reactive antibodies in lysates of cells of the indicated genotype. The cells were untreated or exposed to the protein synthesis inhibitor, cycloheximide (Cx, 100 μg/ml) for the indicated time. In the panel on the right are immunoblots of samples immunoprecipitated with a BiP-specific antiserum from the lysates of the samples shown on the left.

C Immunoblots as in (B) in lysates of cells of the indicated genotype. The cells were untreated or exposed to the protein synthesis inhibitor, cycloheximide (Cx, 100 μg/ml) and/or thapsigargin (Tg) for the indicated periods prior to harvest.

D Quantification of ratio of the BiP-AMP to total BiP signal (expressed in arbitrary units) of repeats of the experiment shown in (C). Mean ± SD of the BiP-AMP signal normalised to total BiP signal from the same samples are depicted (*P* values by two tailed *t*-test, $n = 6$, biological replicates).

BiP, the only Hsp70 chaperone in the mammalian ER, is essential. Complete inactivation of the encoding gene compromises both unicellular eukaryotes such as budding yeast (Normington *et al*, 1989) and metazoans at an early developmental stage (Luo *et al*, 2006). Reduction in BiP levels by exposure to SubA, a bacterial toxin that cleaves the protein intracellularly, is sufficient to

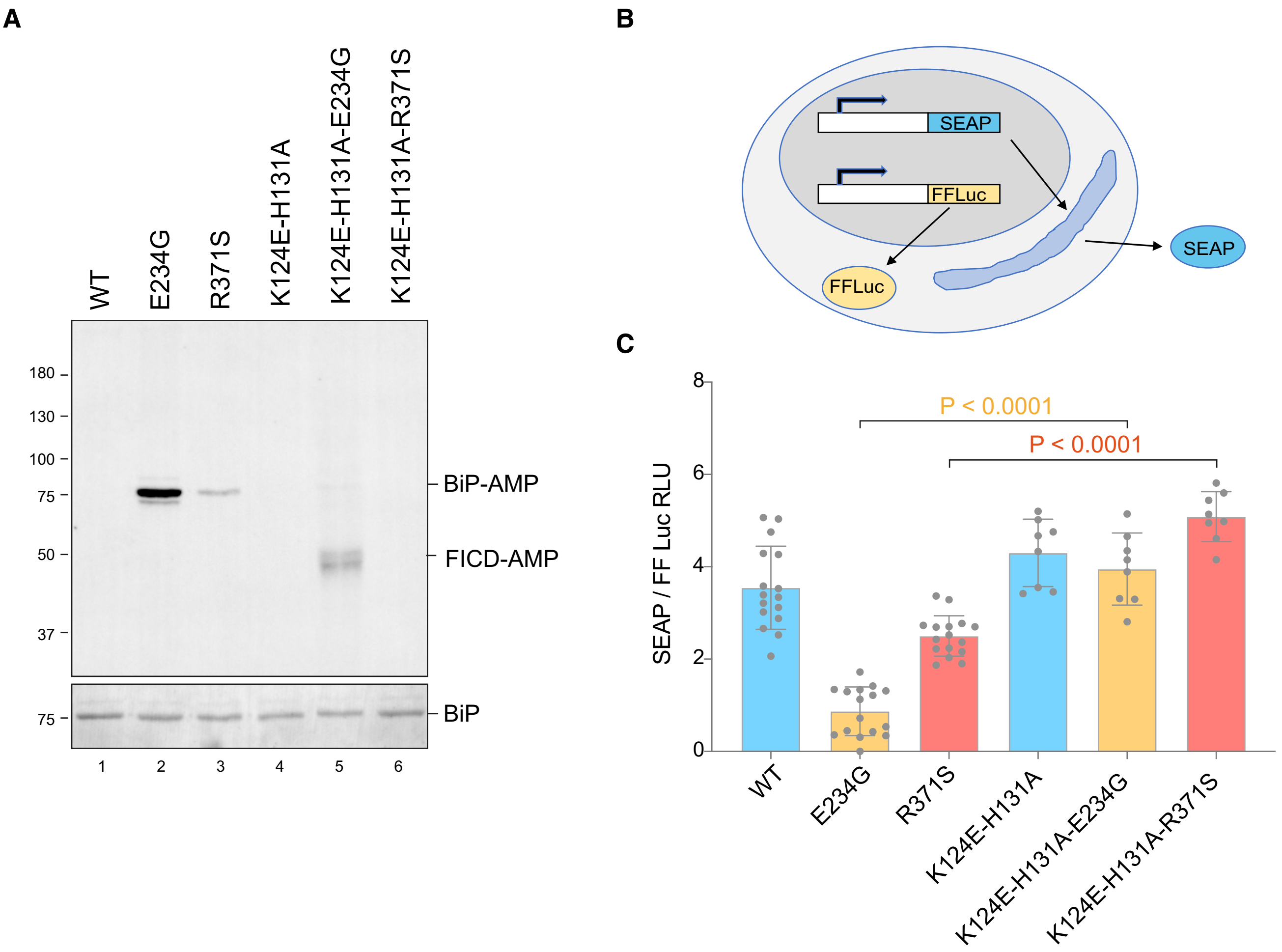

Figure 6. Compromised secretion by deregulated FICD depends on residues specifying BiP AMPylation.

A Representative immunoblot of AMPylated proteins in $FICD^{\Delta}$ CHO cells transiently transfected with expression plasmids encoding the indicated FICD enzymes (replicated three times). Note that the hyperactive E234G mutant bearing the K124E-H131A mutations that compromise its ability to interact with BiP, is nonetheless consistently auto-AMPylated.

B Schema of the assay used to measure the effect of FICD on secretion.

C Bar diagram of the amount of alkaline phosphatase (normalised to cytoplasmic luciferase [a transfection marker]) secreted from $FICD^{\Delta}$ CHO cells co-expressing the indicated FICD enzymes. The K124E-H131A mutations that interfere with engagement of BiP as a substrate reverse the secretion defect imposed by the hyperactive FICD enzymes (Mean ± SD, *P* values by ANOVA with Šídák's multiple comparisons test, $n = 8$–16 [biological replicates]).

 © 2023 The Authors

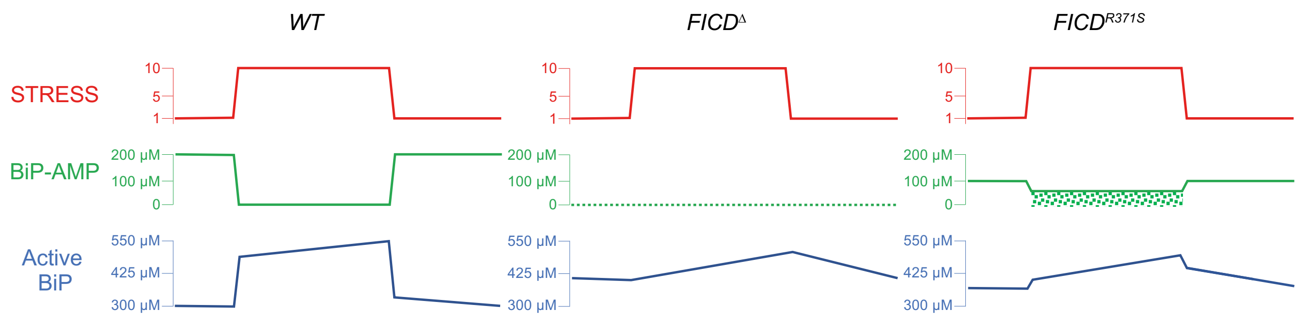

Figure 7. Cartoon contrasting the hypothetical divergent consequences of total absence of FICD (FICD$^{\Delta}$) with the Arg371Ser mutation (FICD^{R371S}) in a pancreatic islet beta cell experiencing a physiologically-driven 10-fold increase in proinsulin biosynthesis.

In wild-type cells (*WT*) the stress imposed by increased production of proinsulin is met by rapid de-AMPylation of BiP and a slow, transcriptionally-mediated increase in BiP synthesis. As the stress wanes, BiP is re-AMPylated followed by slow decline in BiP protein levels, as its biosynthesis decreases. Note, the changes in concentration of BiP-AMP depicted here are based on measurements made in fed and fasted mice (Chambers *et al*, 2012) and assume a total concentration of BiP ~ 500 µM. In *FICD*$^{\Delta}$ cells protein folding homeostasis is presumably maintained by transcriptional mechanisms that are adequate to sustain glycaemic control in knockout mice (McCaul *et al*, 2021). The *FICD*^{R371S} cells are burdened with a residual pool of AMPylated BiP, even when stressed (depicted by stippled green rectangle) compromising ER function.

compromise the secretory apparatus (Paton *et al*, 2006). BiP AMPylation on Thr518, locks the chaperone in an inert conformation that precludes productive engagement of substrate (Wieteska *et al*, 2017; Preissler *et al*, 2017b) and is thus tantamount to a reduction in the concentration of active BiP in the ER.

Normally, this process is subject to tight regulation—surplus BiP is inactivated by AMPylation as ER stress wanes and the reserve pool of AMPyated BiP is reactivated, through deAMPylation, as stress levels rise (reviewed in: Preissler & Ron, 2019; Perera & Ron, 2022). The Arg371Ser mutation compromises both aspects of BiP regulation: timely BiP AMPylation and deAMPylation. The defect in AMPylation capacity explains the diminished ability of mutant cells to rapidly inactivate excess BiP upon a pharmacological reduction in unfolded protein flux into the ER (Fig 7).

The complete absence of deAMPylation activity of the mutant enzyme creates an intrinsic imbalance that favours AMPylation over deAMPylation to deprive cells of a readily accessible pool of dormant BiP when needed. Such deregulation is suggested by a consistent basal level of AMPylated BiP in *FICD*^{R371S} cells, whereas the basal signal in wild-type cells varies. Importantly, under stress conditions wild-type cells can fully re-activate pre-existing pools of AMPylated chaperone, whilst the mutant cells are impaired in this recruitment mechanism (compare the effects of thapsigargin treatment on wild-type and mutant cells in Fig 5).

This scenario is consistent with the recessive nature of the genetic disease. Whilst the imbalance in antagonistic enzymatic activities is intrinsic to the FICD^{R371S} mutant enzyme, our *in vitro* observations suggest that the product of a wild-type *FICD* allele, a powerful deAMPylase (Preissler *et al*, 2017a), can buffer the weak AMPylation bias imposed by the product of the mutant allele (Fig 3F). The buffering task of the wild-type protein in heterozygotes is likely favoured not only by the weak AMPylation activity of FICD^{R371S} but also by the fact that the mutation creates a cryptic glycosylation site in the enzyme's active site. Glycosylation on Asn369 is likely to compromise FICD folding and reduce the burden of AMPylation even further in the heterozygous state. By contrast, in the homozygous state, the weak AMPylation activity of FICD^{R371S} molecules that escape co-translational glycosylation is unopposed.

High levels of AMPylation can inactivate enough BiP to compromise ER function (Casey *et al*, 2017; Moehlman *et al*, 2018) and to trigger a measurable increase in signalling in the ER unfolded protein response (UPR) (Preissler *et al*, 2015b). By contrast, the activities of fluorescent UPR reporters are unaltered under basal conditions in FICD^{R371S}-expressing cells. This likely reflects their adaptation to the mutant allele and suggests that the consequences of deregulated AMPylation are not manifest in the basal state but rather as cells experience fluctuations in the burden of ER client proteins. Insulin-producing beta cells are known to face large (glucose-driven) excursions in the burden of unfolded pro-insulin entering their ER (> 10 fold increase over 30 min, Itoh & Okamoto, 1980). A defect in recruiting AMPylated BiP to the chaperone cycle as beta cells cope with physiological glycaemic excursions, may account for their sensitivity to this mutation.

Whilst this paper was under review a report appeared describing five individuals with a slowly progressive neurodegenerative disorder of the motor neurons resulting from homozygosity of a specific, different mutation in FICD's active site—p.(Arg374His) (Rebelo *et al*, 2022). The p.(Arg374His) mutant protein was not characterised *in vitro* but patient-derived fibroblasts exhibited elevated levels of BiP-AMP, consistent with a defect that favours AMPylation over deAMPylation. Thus, two different mutations that bias FICD towards BiP hyper-AMPylation compromise the nervous system, albeit with important differences in the phenotype and age of onset. Importantly, the Arg371Ser mutation identified in our patients resulted in a multi-system disease, including bilateral cataracts, deafness and infancy-onset diabetes. Whilst the basis for the phenotypic differences resulting from the two mutations is currently not understood, the genetic evidence suggests mutation-specific mechanisms resulting in distinct FICD-related diseases.

It is interesting to contrast the consequences of complete loss of FICD function, which has no effect on glycaemic control or gross neurological function in mice (McCaul *et al*, 2021; Casey *et al*, 2022), with the homozygous human p.(Arg371Ser) and p.(Arg374His) mutations. From a mechanistic perspective, this suggests that loss of AMPylation/deAMPylation as a post-translational strand of the UPR can be buffered by parallel regulatory processes,

whilst inability to antagonise constitutive AMPylation is poorly tolerated (Fig 7). It also implies that the profound physiological perturbation arising from homozygosity of the *FICD*^{R371S} mutation could be reversed by complete inactivation of FICD, for example by a small molecule that blocks the enzyme's adenosine-binding site.

Here, we interpret the consequences of the FICD^{R371S} mutation through the inactivating features of BiP AMPylation: assuming that BiP-AMP is completely inert, and that the phenotype arises from a deficit of active chaperone. However, it remains possible that BiP-AMP retains important biochemical activities whose deregulation contributes to the phenotype via biochemical gain-of-function features. For example, it is possible that constitutive AMPylation of BiP may result in inappropriate sequestration of BiP co-chaperones (away from the active/unmodified pool of BiP).

Our focus on BiP is justified by the observation that it is the main protein to undergo detectable incorporation of AMP (fig 1 in Carlsson & Lazarides, 1983 and 17G6 immunoblots here). However, it is noteworthy that the therapeutic strategy articulated above is relevant even if the pathogenic consequences of the mutation were to be realised via deregulated AMPylation of other, yet to be discovered, FICD substrates. Finally, it is intriguing to consider that the severe consequences of a genetic disease, which highlights the cost of an extreme imbalance in BiP AMPylation, may hint at the therapeutic utility of re-balancing the AMPylation/deAMPylation poise of wild-type FICD in other extreme circumstances.

Materials and Methods

Table EV2 List the plasmids and Table EV3 the key resources used in this study

Subjects

Individuals with diabetes diagnosed before the age of 12 months were recruited by their clinicians for molecular genetic analysis in the Exeter Molecular Genetics Laboratory. The study was conducted in accordance with the Declaration of Helsinki, the United States Department of Health and Human Services Belmont Report. All subjects or their parents/guardians gave informed consent for genetic testing.

Genetic analysis

Genome sequencing was performed on DNA extracted from peripheral blood leukocytes. The samples from individuals 1a and 1b were sequenced on an Illumina HiSeq X10 with a mean read depth of 41.2 and 38.3, respectively. The samples of 126 individuals were analysed by whole-genome sequencing on an Illumina HiSeq 2,500 (n = 8), HiSeq X10 (n = 71) or with BGISeq-500 (n = 47). The sequencing data were analysed using an approach based on the GATK best practice guidelines. GATK haplotypecaller was used to identify variants which were annotated using Alamut batch version 1.11 (Sophia Genetics) and variants which failed the QD2 VCF filter or had < 5 reads supporting the variant allele were excluded. Copy number variants were called by SavvyCNV (Laver *et al*, 2022) which uses read depth to judge copy number states. SavvyVcfHomozygosity (https://github.com/rdemolgen/SavvySuite) was used to identify large (> 3 Mb) homozygous regions in the genome sequencing data. An in-house software was used to detect shared haplotypes (https://github.com/rdemolgen/SavvySuite).

Protein purification

The structured regions of human FICD proteins (residues 104–445) and BiP$^{T229A\text{-}V461F}$ (residues 27–635) were expressed in T7 Express *lysY/I*^{q} (NEB) *E. coli* cells as N-terminal His$_6$-SUMO fusion proteins or as GST fusion proteins. The proteins were expressed and purified (Perera *et al*, 2019, 2021). In brief, the His6-SUMO tag was removed from the FICD/BiP fusion partner by Ulp1 cleavage following Ni-NTA affinity purification or the GST tag by TEV protease cleavage. The cleaved proteins of interest were further purified by anion exchange (using a RESOURCE Q 6 ml column [Cytivia]) and gel filtration (using a S200 Increase 10/300 GL column [Cytivia]). Proteins were concentrated in a final buffer consisting of 25 mM Tris pH 8.0, 150 mM NaCl and 1 mM TCEP (buffer TNT). The plasmids used to express the FICD and BiP proteins utilised in this study are detailed in Table EV3.

Differential scanning fluorimetry (DSF)

DSF analyses were conducted as in (Perera *et al*, 2019, 2021) with minor modifications: final DSF samples contained 1 μM FICD protein ± 2.5 mM nucleotide (as indicated in Fig 3A) in a buffer of HKM supplemented with 1.5× SYPRO Orange protein gel stain (Thermo Fisher Scientific).

Analytical size exclusion chromatography

Analytical size exclusion chromatography was conducted on an Agilent Bio SEC-3 HPLC column (300 Å pore size, 3 μm particle size, 4.6 × 300 mm) equilibrated in TNT buffer at 25°C. The FICD proteins were diluted in TNT buffer to the indicated concentration (Fig 3B) and incubated for at least 1 h at room temperature before a 10 μl volume of the protein solution was injected (using an HPLC autosampler) onto the column. Protein was eluted at a flow rate of 0.3 ml/min.

In vitro AMPylation assay

5 μM unmodified BiP substrate was incubated at 25°C with FICD proteins at the indicated concentration for the indicated time (Fig 3C and F) in a buffer consisting of HKM (25 mM HEPES-KOH pH 7.4, 150 mM KCl and 10 mM $MgCl_2$) supplemented with 5 mM ATP. Reactions were quenched by the addition of LDS-PAGE sample buffer and heating to 70°C. Samples containing 1 μg of BiP were loaded onto and resolved on a 4–12% SDS–PAGE gel and subsequently wet-transferred onto a PVDF membrane. Total protein was imaged using Ponceau S stain (Fig 3C) or by use of parallel gels visualised with Coomassie protein stain (Fig 3F). The membrane was blocked with 1× ROTI Block (Roth) diluted in water and then probed for 1 h at 25°C with a mouse monoclonal IgG antibody reactive to AMPylated proteins (MoAb 17G6, Hopfner *et al*, 2020), diluted 1/1000 (v/v) in 1 × ROTI Block. The AMPylated BiP signal was imaged using an IRDye 800CW goat anti-mouse IgG secondary antibody [Li-Cor], diluted 1/2000 (v/v) in 1x ROTI-Block.

© 2023 The Authors

In vitro deAMPylation assay

The fluorescence anisotropy-based deAMPylation assay and the k_{cat} calculation (for wild-type FICD) was conducted as in Perera *et al* (2019, 2021) with minor modifications. Namely, each deAMPylation reaction was carried out in a 15 μl volume containing 100 μM $BiP^{T229A-V461F}$-AMP (a concentration previously found to be able to saturate wild-type FICD) and 10 nM $BiP^{T229A-V461F}$ modified with FAM labelled AMP, in a buffer of HKM supplemented with 0.05% (*v*/*v*) Triton X-100. The assay was conducted in a 384-well non-binding, low volume, HiBase, black microplate (greiner bio-one). Wild-type and Arg371Ser FICD enzymes were added at $t = 0$ to a final concentration of 10 μM. Note, $FICD^{R371S}$ enzyme at 20 μM also failed to catalyse any discernible deAMPylation.

The fluorescence anisotropy of the FAM signal was recorded on a CLARIOstar Plus plate reader (BMG Labtech) exciting at 482–16 nm and top reading emission at 530–40 nm. A reference well containing only 10 nM N^6-(6-Amino)hexyl-ATP-6-FAM (Jena Bioscience) was used to set the relative gains in the parallel and perpendicular emission channels (targeting 25 mP units). The deAMPylation time course was conducted with the CLARIOstar maintaining a temperature of 25°C, whilst also mixing the sample plate using a 2 s double orbital shake after each kinetic cycle. The fluorescence anisotropy signal of fully deAMPylated BiP was estimated from the plateau value of a single exponential decay curve heuristically fitted to the deAMPylation trace catalysed by wild-type FICD.

Mammalian cell culture

The previously described cell lines CHO-K1, CHO-K1-$FICD^{\Delta}$ (Preissler *et al*, 2015b) and CHO-K1-$FICD^{R371S}$ cells (described below and in Key Resource Table) were cultured in Nutrient mixture F-12 Ham (Sigma) supplemented with 10% (vol/vol) serum (FetalClone II; HyClone), 1× Penicillin–Streptomycin (Sigma) and 2 mM L-glutamine (Sigma) at 37°C and 5% CO_2. The identity of the CHO cell lines has been authenticated using the criteria of: A. successful targeting of essential genes using species-specific CRISPR whole genome library, and B. sequencing of the wildtype or mutant alleles of the genes studied that confirmed the sequence reported for the corresponding genome. The cell lines tested negative for mycoplasma contamination using a commercial kit (MycoAlert (TM) Mycoplasma Detection Kit, Lonza). None of the cell lines is on the list of commonly misidentified cell lines maintained by the International Cell Line Authentication Committee.

FICD mutation knock-in

Plasmid and oligonucleotide reagents referenced herein are listed in Tables EV2 and EV3—plasmid list and Key Resources Tables respectively. A guide targeting *Ficd* was selected in the region of Exon 2 (previously called exon 3) surrounding the codon for R371S into the CCTop—CRISPR/Cas9 target online predictor (Stemmer *et al*, 2015) and duplex DNA oligonucleotides (3035_g2_R371_S, 3036_g2_R371_AS) of the sequence was inserted into the pSpCas9 (BB)-2A-mCherry_V2 plasmid (UK1610) to create guide plasmid UK2959. Cells in 80% confluent 6-well dishes were transfected 24 h post plating with 0.5 μg guide plasmid (UK2959) and 1.5 μg of a single-stranded oligonucleotide repair template (3037_FICD_R371S_repair_V2S) that contains base changes to introduce the R371S allele as well as silent mutations introducing a *Bam*HI site and pam destroying mutation using Lipofectamine LTX (Invitrogen). Thirty-six hours post-transfection the cells were washed with PBS, resuspended in PBS containing 4 mM EDTA and 0.5% (w/v) BSA, and mCherry-positive cells were individually sorted by fluorescence-activated cell sorting (FACS) into 96-well plates using a Melody Cell sorter (Beckman Coulter). Genomic DNA was isolated from 186 clones of which 171 produced a 296 bp PCR product with primers 3003_cgFICD_F and 3004_cgFICD_R. Upon digestion with *Bam*HI, 5 had the expected size fragments and sequencing of these identified a single clone (R371S-C74) with a single correct R371S mutant allele.

Cell treatment, transfection and analysis

Cells (80% confluent) were transfected 24 h after plating with 2 μg of the indicated expression plasmids per well of a 6-well (35 cm) dish using Lipofectamine LTX (Life Technologies) according to manufacturer's instructions. The medium was exchanged after 24 h and harvest for Flow cytometry or immunoblot were performed 36 h after transfection (Fig 4). For analysis of CHO wildtype, $FICD^D$ or $FICD^{R371S}$ cells, medium on 80% confluent 10 cm dishes of cells was exchanged 1 h prior to drug treatments of cells (100 μg/ml cycloheximide or 0.2 μM thapsigargin) of the indicated genotype for 3 or 6 h prior to harvest as indicated in the figures.

For immunoblotting cells were washed twice in PBS-2 mM EDTA and collected in the same and then lysed as previously described (Preissler *et al*, 2015a) in 4 cell volumes HG buffer (50 mM HEPES–KOH pH 7.4, 150 mM NaCl, 2 mM MgCl2, 33 mM D-glucose, 10% [v/v] glycerol, 1% [v/v]) Triton X-100 and protease inhibitors (2 mM phenylmethylsulphonyl fluoride [PMSF], 4 μg/ml pepstatin, 4 μg/ml leupeptin, 8 μg/ml aprotinin) with 100 U/ml hexokinase (from Saccharomyces cerevisiae Type F-300; Sigma). Lysates were cleared at 20,000 g and 20 μg of protein was separated by PAGE and transferred to PDVF membranes. For Immunoprecipitation, 430 μg of lysate was precleared with 20 μl UltraLink Hydrazine Resin (Pierce cat. # 53149) blocked with aniline for 1 h at 4C followed by incubation with 20 μl UltraLink Hydrazine Resin on which BiP-specific chicken IgY antibodies have been covalently immobilised according to the manufacturer's instructions (Preissler *et al*, 2015a). After washing three times with lysis buffer the proteins were eluted with Laemmli buffer prior to PAGE and transfer to PDVF. The PVDF membranes were subsequently blocked in 1/10 dilution of ROTI®Block 10×, 5% BSA in TBS (50 mM Tris–Cl, pH 7.5) then sequentially incubated with primary antisera diluted in blocking buffer as follows: anti-AMP (MoAb 17G6) and eIF2α [mouse anti-eIF2α (Avezov *et al*, 2013)] hamster BiP [chicken anti-hamster BiP (Avezov *et al*, 2013)], and FICD [chicken anti-FICD (Preissler *et al*, 2015b)] were used at a dilutions of 1/1,000, 1/5,000 and 1/1,000, and 1/1,000 (v/v), respectively. The membranes were then washed 3 times in TBS and incubated with secondary antisera linked to IR800 (1/2,000 v/v) or Cy3 (1/1,000 v/v) in blocking buffer. The membranes were scanned with an Odyssey near-infrared imager (LI-COR) to detect IR800 secondary antisera or Biorad ChemiDocTM MP Imaging system with v3.0.1.14 Image Lab

© 2023 The Authors

Touch software to detect Cy3. Where applicable, IB band quantification was carried out using NIH-Image (Fiji) Gel tool.

Secreted alkaline phosphatase (SEAP) assay

Cells were transfected with 500 ng FICD expression vector, 100 ng SEAP expression vector (UK1014), and 25 ng SV40_Luc_pGL3 expression vector (UK 3075, a transfection marker) per well in 24 well dishes of FICD−/−10 CHO cells. 16 h later the medium was changed and after a further 24 h of culture the medium was collected, heated at 60°C to for 1 h prior to assay for SEAP activity at room temperature by mixing 20 µl of heat-treated medium to 100 µl 1 M diethanolamine buffer, pH 9.8, 0.5 mM MgCl2 containing 1 mg/ml freshly dissolved phosphatase substrate (4-Nitrophenyl phosphate disodium salt hexahydrate, Sigma, S0942) and measuring the OD405 and OD 630 every 4.75 min for 20 cycles. The cells were lysed in the dish in 250 µl luciferase lysis buffer (25 mM gly-gly, 15 mM $MgSO_4$, 4 mM EGTA, 1 mM DTT, 1% Triton X 100) for 20 min on ice and 25 µl was assayed for luciferase activity by addition of 25 µl of luciferase assay reagent (25 mM gly-gly, 15 mM MgSO4, 4 mM EGTA, 11.7 mM potassium phosphate, 1.6 mM ATP Sigma A2383, 0.2 mg/ml coenzyme A Alfa-Aesar J65434.MC, 500 µM d-Luciferin, ABcam ab143655) in a BMG labtech Clariostar plate reader using SMART control v 6.10 acquisition software and MARS v 4.10 data analysis software. ANOVA in Prism software indicated that there was a significant difference amongst means ($P < 0.0001$) and Šídák's multiple comparisons test was used to determine the significance between data pairs as indicated in the legend. Parallel 6 well dishes were transfected and harvested for immunoblot as in Figs 4 and 5.

Statistical analysis

The statistical tools used in the genetic analysis are described above (in Genetic analysis) and listed in Table EV3. Statistical analysis of the biochemical and cellular data was performed using GraphPad Prism 9.4.1 (GraphPad Software Inc.). Statistical significance between groups was calculated using unpaired Student's *t*-test with *P*-values < 0.05 considered statistically significant.

The paper explained

Problem
Diabetes mellitus in humans commonly develops in the context of resistance to insulin action (type II) or an immune assault on the insulin-producing beta cells (type I). By contrast Diabetes mellitus presenting soon after birth (neonatal diabetes) is often caused by mutations in genes that compromise the machinery of insulin-producing beta cells. The genetic study of families with these rare subtypes of diabetes provides important insight into pathogenetic mechanisms operating at the level of beta cells. These processes, in less extreme forms, may also contribute to the more common types of diabetes and their understanding may provide insight into potential therapies.

Results
Genome sequencing led to the discovery of five individuals, from three families, with onset of diabetes in the first year of life, severe neurodevelopmental delay, and homozygosity for the same mutation (p.Arg371Ser) in the gene encoding FICD. Genetic analysis provides strong evidence for the causal relationship between this mutation and the disease. FICD is an enzyme that catalyses two antagonistic reactions: The conjugation of adenosine monophosphate (AMP) to the endoplasmic reticulum (ER) chaperone BiP, to inactivate BiP, and reactivating removal of the AMP moiety. Normally, FICD carries out these activities in a highly regulated manner, responding to a cell's need for active BiP. The Arg371Ser mutation derails this process. Given BiP's important role in the folding of secreted proteins, including insulin, it seems plausible that inappropriately high levels of AMPylated BiP contribute to impaired secretion and dysfunction of beta cells and cells relevant to neurodevelopment.

Clinical Impact
Identification of human disease caused by deregulated activity of an ER-localised AMPylase expands the inventory of biochemical processes known to be required for ER homeostasis in health. Importantly, analysis of this rare genetic change also suggests the possibility of a specific remedy. Whilst FICD deregulation by the Arg371Ser mutation is severely pathogenic, complete loss of FICD has only minimal consequences (in mice). Thus, it stands to reason that an inhibitor that blocks all FICD activity may be useful in treating individuals homozygous for the Arg371Ser mutation, as the underlying pathophysiological process relies on deregulated AMPylation.

Data availability

This study includes no data deposited in external repositories.

Expanded View for this article is available online.

Acknowledgements
We thank Dorothea Hopfner and Aymelt Itzen (Hamburg-Eppendorf University) for the generous gift of the 17G6 monoclonal antibody to AMP, Dr Richard Caswell (Exeter Genomics laboratory) for helpful discussion regarding the mutation mechanism, the CIMR flow cytometry core (Reiner Schulte, Chiara Cossetti, and Gabriela Grondys-Kotarba) for help with FACS measurements. Supported by a Wellcome Trust Principal Research Fellowship to D.R. (Wellcome 224407/Z/21Z). A Diabetes UK RD Lawrence fellowship to E.D.F. (Grant number 19_0005971), and a Wellcome Trust Senior Research Fellowship to S.E.F. (105636/Z/14/Z). M.N.W. is in receipt of an Independent Fellowship from the Exeter Diabetes Centre of Excellence funded by Research England's Expanding Excellence in England (E3) fund. For the purpose of open access, the authors have applied a Creative Commons Attribution (CC BY) licence to any Author Accepted Manuscript version arising from this submission.

Author contributions
Luke A Perera: Conceptualization; investigation; methodology; writing—original draft; writing—review and editing. **Andrew T Hattersley:** Conceptualization; supervision; funding acquisition; methodology; writing—review and editing. **Heather P Harding:** Conceptualization; investigation; methodology; writing—review and editing. **Matthew N Wakeling:** Data curation; formal analysis; methodology. **Sarah E Flanagan:** Data curation; formal analysis; methodology. **Ibrahim Mohsina:** Resources. **Jamal Raza:** Resources. **Alice Gardham:** Resources. **David Ron:** Conceptualization; supervision; funding acquisition; investigation; methodology; writing—original draft; project administration; writing—review and editing. **Elisa De Franco:** Conceptualization; data curation; formal analysis; funding acquisition; investigation; methodology; writing—original draft; project administration; writing—review and editing.

 © 2023 The Authors

Disclosure and competing interests statement

The authors declare that they have no conflict of interest.

For more information

The Ron Lab website <https://ron.cimr.cam.ac.uk>.

Monogenic diabetes research centre at the University of Exeter <https://www.diabetesgenes.org/about-neonatal-diabetes/>.

References

Avezov E, Cross BC, Kaminski Schierle GS, Winters M, Harding HP, Melo EP, Kaminski CF, Ron D (2013) Lifetime imaging of a fluorescent protein sensor reveals surprising stability of ER thiol redox. *J Cell Biol* 201: 337 – 349

Braakman I, Hebert DN (2013) Protein folding in the endoplasmic reticulum. *Cold Spring Harb Perspect Biol* 5: a013201

Bunney TD, Cole AR, Broncel M, Esposito D, Tate EW, Katan M (2014) Crystal structure of the human, FIC-domain containing protein HYPE and implications for its functions. *Structure* 22: 1831 – 1843

Carlsson L, Lazarides E (1983) ADP-ribosylation of the Mr 83,000 stress-inducible and glucose-regulated protein in avian and mammalian cells: modulation by heat shock and glucose starvation. *Proc Natl Acad Sci USA* 80: 4664 – 4668

Casey AK, Moehlman AT, Zhang J, Servage KA, Kramer H, Orth K (2017) Fic-mediated deAMPylation is not dependent on homodimerization and rescues toxic AMPylation in flies. *J Biol Chem* 292: 21193 – 21204

Casey AK, Gray HF, Chimalapati S, Hernandez G, Moehlman AT, Stewart N, Fields HA, Gulen B, Servage KA, Stefanius K *et al* (2022) Fic-mediated AMPylation tempers the unfolded protein response during physiological stress. *Proc Natl Acad Sci USA* 119: e2208317119

Chambers JE, Petrova K, Tomba G, Vendruscolo M, Ron D (2012) ADP ribosylation adapts an ER chaperone response to short-term fluctuations in unfolded protein load. *J Cell Biol* 198: 371 – 385

Danilova T, Belevich I, Li H, Palm E, Jokitalo E, Otonkoski T, Lindahl M (2019) MANF is required for the postnatal expansion and maintenance of pancreatic beta-cell mass in mice. *Diabetes* 68: 66 – 80

Delepine M, Nicolino M, Barrett T, Golamaully M, Lathrop GM, Julier C (2000) EIF2AK3, encoding translation initiation factor 2-alpha kinase 3, is mutated in patients with Wolcott-Rallison syndrome. *Nat Genet* 25: 406 – 409

Engel P, Goepfert A, Stanger FV, Harms A, Schmidt A, Schirmer T, Dehio C (2012) Adenylylation control by intra- or intermolecular active-site obstruction in fic proteins. *Nature* 482: 107 – 110

Fauser J, Gulen B, Pogenberg V, Pett C, Pourjafar-Dehkordi D, Krisp C, Hopfner D, Konig G, Schluter H, Feige MJ *et al* (2021) Specificity of AMPylation of the human chaperone BiP is mediated by TPR motifs of FICD. *Nat Commun* 12: 2426

Ham H, Woolery AR, Tracy C, Stenesen D, Kramer H, Orth K (2014) Unfolded protein response-regulated drosophila fic (dFic) protein reversibly AMPylates BiP chaperone during endoplasmic reticulum homeostasis. *J Biol Chem* 289: 36059 – 36069

Hendershot LM, Ting J, Lee AS (1988) Identity of the immunoglobulin heavy-chain-binding protein with the 78,000-Dalton glucose-regulated protein and the role of posttranslational modifications in its binding function. *Mol Cell Biol* 8: 4250 – 4256

Hopfner D, Fauser J, Kaspers MS, Pett C, Hedberg C, Itzen A (2020) Monoclonal anti-AMP antibodies are sensitive and valuable tools for detecting patterns of AMPylation. *iScience* 23: 101800

Itoh N, Okamoto H (1980) Translational control of proinsulin synthesis by glucose. *Nature* 283: 100 – 102

Karczewski KJ, Francioli LC, Tiao G, Cummings BB, Alfoldi J, Wang Q, Collins RL, Laricchia KM, Ganna A, Birnbaum DP *et al* (2020) The mutational constraint spectrum quantified from variation in 141,456 humans. *Nature* 581: 434 – 443

Khater S, Mohanty D (2015) In silico identification of AMPylating enzymes and study of their divergent evolution. *Sci Rep* 5: 10804

Kinch LN, Yarbrough ML, Orth K, Grishin NV (2009) Fido, a novel AMPylation domain common to fic, doc, and AvrB. *PLoS One* 4: e5818

Kingdon HS, Shapiro BM, Stadtman ER (1967) Regulation of glutamine synthetase. 8. ATP: glutamine synthetase adenylyltransferase, an enzyme that catalyzes alterations in the regulatory properties of glutamine synthetase. *Proc Natl Acad Sci USA* 58: 1703 – 1710

Laitusis AL, Brostrom MA, Brostrom CO (1999) The dynamic role of GRP78/BiP in the coordination of mRNA translation with protein processing. *J Biol Chem* 274: 486 – 493

Laver TW, De Franco E, Johnson MB, Patel KA, Ellard S, Weedon MN, Flanagan SE, Wakeling MN (2022) SavvyCNV: genome-wide CNV calling from off-target reads. *PLoS Comput Biol* 18: e1009940

Luo S, Mao C, Lee B, Lee AS (2006) GRP78/BiP is required for cell proliferation and protecting the inner cell mass from apoptosis during early mouse embryonic development. *Mol Cell Biol* 26: 5688 – 5697

McCaul N, Porter CM, Becker A, Tang CA, Wijne C, Chatterjee B, Bousbaine D, Bilate A, Hu CA, Ploegh H *et al* (2021) Deletion of mFICD AMPylase alters cytokine secretion and affects visual short-term learning *in vivo*. *J Biol Chem* 100991: 100991

Moehlman AT, Casey AK, Servage K, Orth K, Kramer H (2018) Adaptation to constant light requires fic-mediated AMPylation of BiP to protect against reversible photoreceptor degeneration. *Elife* 7: e38752

Normington K, Kohno K, Kozutsumi Y, Gething MJ, Sambrook J (1989) *S. cerevisiae* encodes an essential protein homologous in sequence and function to mammalian BiP. *Cell* 57: 1223 – 1236

Paton AW, Beddoe T, Thorpe CM, Whisstock JC, Wilce MC, Rossjohn J, Talbot UM, Paton JC (2006) AB5 subtilase cytotoxin inactivates the endoplasmic reticulum chaperone BiP. *Nature* 443: 548 – 552

Perera LA, Ron D (2022) AMPylation and endoplasmic reticulum protein folding homeostasis. *Cold Spring Harb Perspect Biol* 30: a041265

Perera LA, Rato C, Yan Y, Neidhardt L, McLaughlin SH, Read RJ, Preissler S, Ron D (2019) An oligomeric state-dependent switch in the ER enzyme FICD regulates AMPylation and deAMPylation of BiP. *EMBO J* 38: e102177

Perera LA, Preissler S, Zaccai NR, Prevost S, Devos JM, Haertlein M, Ron D (2021) Structures of a deAMPylation complex rationalise the switch between antagonistic catalytic activities of FICD. *Nat Commun* 12: 5004

Pobre KFR, Poet GJ, Hendershot LM (2019) The endoplasmic reticulum (ER) chaperone BiP is a master regulator of ER functions: getting by with a little help from ERdj friends. *J Biol Chem* 294: 2098 – 2108

Preissler S, Ron D (2019) Early events in the endoplasmic reticulum unfolded protein response. *Cold Spring Harb Perspect Biol* 11: a033894

Preissler S, Chambers JE, Crespillo-Casado A, Avezov E, Miranda E, Perez J, Hendershot LM, Harding HP, Ron D (2015a) Physiological modulation of BiP activity by *trans*-protomer engagement of the interdomain linker. *Elife* 4: e08961

Preissler S, Rato C, Chen R, Antrobus R, Ding S, Fearnley IM, Ron D (2015b) AMPylation matches BiP activity to client protein load in the endoplasmic reticulum. *Elife* 4: e12621

© 2023 The Authors

Preissler S, Rato C, Perera LA, Saudek V, Ron D (2017a) FICD acts bifunctionally to AMPylate and de-AMPylate the endoplasmic reticulum chaperone BiP. *Nat Struct Mol Biol* 24: 23–29
Preissler S, Rohland L, Yan Y, Chen R, Read RJ, Ron D (2017b) AMPylation targets the rate-limiting step of BiP's ATPase cycle for its functional inactivation. *Elife* 6: e29428
Rahman M, Ham H, Liu X, Sugiura Y, Orth K, Kramer H (2012) Visual neurotransmission in drosophila requires expression of fic in glial capitate projections. *Nat Neurosci* 15: 871–875
Rebelo AP, Ruiz A, Dohrn MF, Wayand M, Farooq A, Danzi MC, Beijer D, Aaron B, Vandrovcova J, Houlden H *et al* (2022) BiP inactivation due to loss of the deAMPylation function of FICD causes a motor neuron disease. *Genet Med* 24: 2487–2500
Sanchez Caballero L, Gorgogietas V, Arroyo MN, Igoillo-Esteve M (2021) Molecular mechanisms of beta-cell dysfunction and death in monogenic forms of diabetes. *Int Rev Cell Mol Biol* 359: 139–256
Sanyal A, Chen AJ, Nakayasu ES, Lazar CS, Zbornik EA, Worby CA, Koller A, Mattoo S (2015) A novel link between fic (filamentation induced by cAMP)-mediated adenylylation/AMPylation and the unfolded protein response. *J Biol Chem* 290: 8482–8499
Shrestha N, De Franco E, Arvan P, Cnop M (2021) Pathological beta-cell endoplasmic reticulum stress in type 2 diabetes: current evidence. *Front Endocrinol (Lausanne)* 12: 650158
Stemmer M, Thumberger T, Del Sol Keyer M, Wittbrodt J, Mateo JL (2015) CCTop: an intuitive, flexible and reliable CRISPR/Cas9 target prediction tool. *PLoS One* 10: e0124633
Synofzik M, Haack TB, Kopajtich R, Gorza M, Rapaport D, Greiner M, Schonfeld C, Freiberg C, Schorr S, Holl RW *et al* (2014) Absence of BiP co-chaperone DNAJC3 causes diabetes mellitus and multisystemic neurodegeneration. *Am J Hum Genet* 95: 689–697
Truttmann MC, Cruz VE, Guo X, Engert C, Schwartz TU, Ploegh HL (2016) The Caenorhabditis elegans protein FIC-1 is an AMPylase that covalently modifies heat-shock 70 family proteins, translation elongation factors and histones. *PLoS Genet* 12: e1006023
Truttmann MC, Zheng X, Hanke L, Damon JR, Grootveld M, Krakowiak J, Pincus D, Ploegh HL (2017) Unrestrained AMPylation targets cytosolic chaperones and activates the heat shock response. *Proc Natl Acad Sci USA* 114: E152–E160
Wieteska L, Shahidi S, Zhuravleva A (2017) Allosteric fine-tuning of the conformational equilibrium poises the chaperone BiP for post-translational regulation. *Elife* 6: e29430
Woolery AR, Luong P, Broberg CA, Orth K (2010) AMPylation: something old is new again. *Front Microbiol* 1: 113–119
Worby CA, Mattoo S, Kruger RP, Corbeil LB, Koller A, Mendez JC, Zekarias B, Lazar C, Dixon JE (2009) The fic domain: regulation of cell signaling by adenylylation. *Mol Cell* 34: 93–103
Yarbrough ML, Li Y, Kinch LN, Grishin NV, Ball HL, Orth K (2009) AMPylation of rho GTPases by vibrio VopS disrupts effector binding and downstream signaling. *Science* 323: 269–272
Yong J, Johnson JD, Arvan P, Han J, Kaufman RJ (2021) Therapeutic opportunities for pancreatic beta-cell ER stress in diabetes mellitus. *Nat Rev Endocrinol* 17: 455–467

License: This is an open access article under the terms of the Creative Commons Attribution License, which permits use, distribution and reproduction in any medium, provided the original work is properly cited.

Expanded View Figures

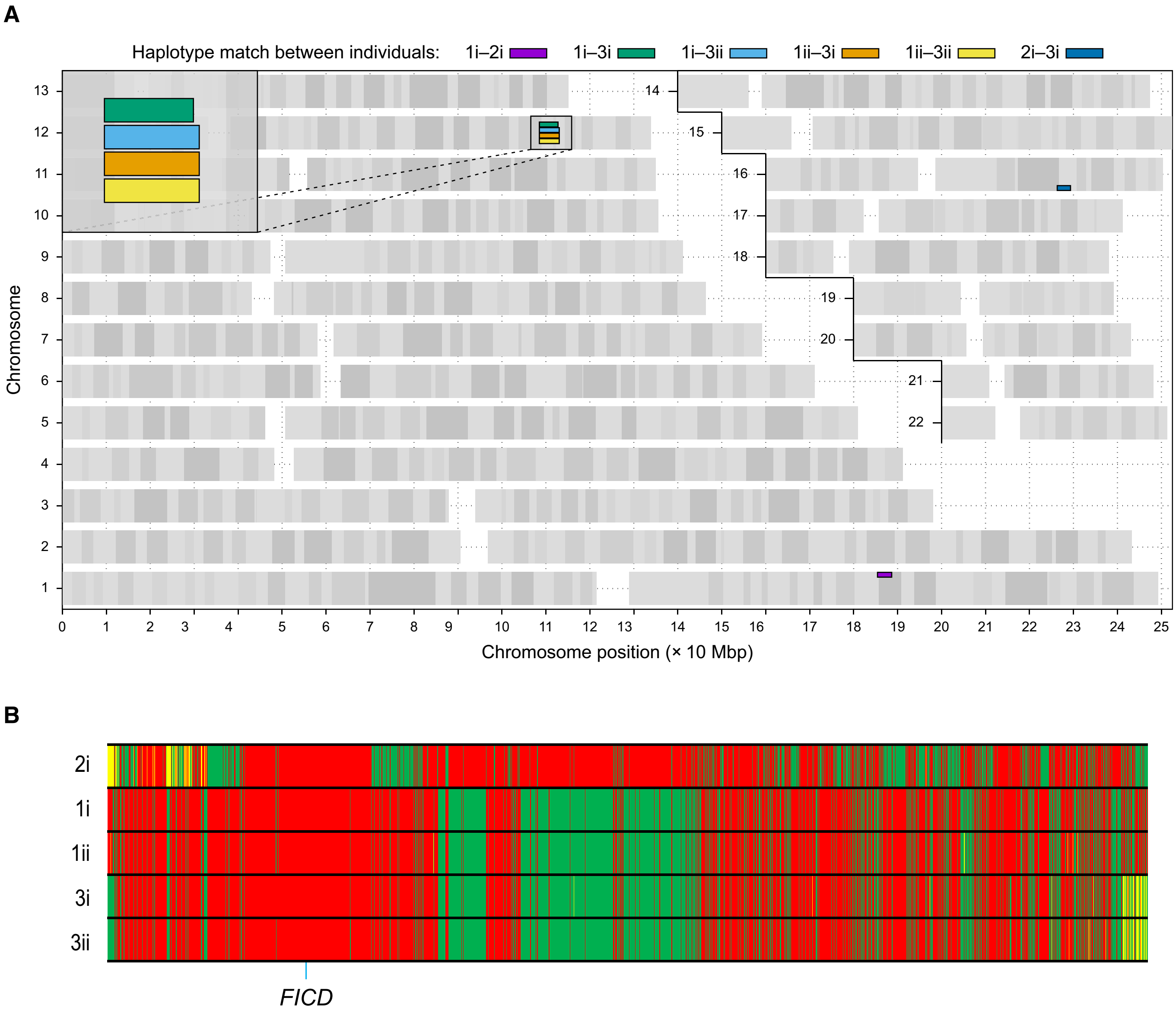

Figure EV1. Analysis of shared haplotypes amongst affected individuals homozygous for the p.(Arg371Ser) mutation in *FICD*.

A Graphical representation of genome-wide shared genomic segments larger than 3 Mbp in the five individuals with *FICD* homozygous mutations. Each colour bar represents a haplotype shared by two individuals (across all four copies of the same chromosome), labelled according to Fig 1A. The four rectangles on the long arm of chromosome 12 (inset) show that the four individuals in Families 1 and 3 share a haplotype [approximate coordinates chr12(hg19):108512514–113023258] including the *FICD* gene [chr12(hg19):108909051–108913380], but none of them shares a haplotype with the patient in Family 2 (individual 2i) —hence the lack of annotated purple or dark blue bars. Note, genotype information was not available for family member 2ii.

B Genotype calls for 2,735 single nucleotide, not multiallelic variants, located on chr12:(hg19):108500000–113100000 in the five individuals with the *FICD* homozygous p.(Arg371Ser) mutation. Only variants where at least one of the five patients carries the alternative allele are shown. Variants with a coverage of < 15 reads and an allele balance for heterozygous calls < 0.25 were removed. The position of the three variants in the *FICD* locus (including the pathogenic p.(Arg371Ser) variant) is indicated at the bottom of the graph. Green = homozygous for the reference allele, Yellow = heterozygous, Red = homozygous for the alternative allele. Note the similarity in variant pattern in affected individuals from families 1 & 3 and the dissimilarity with the affected member of family 2.

© 2023 The Authors

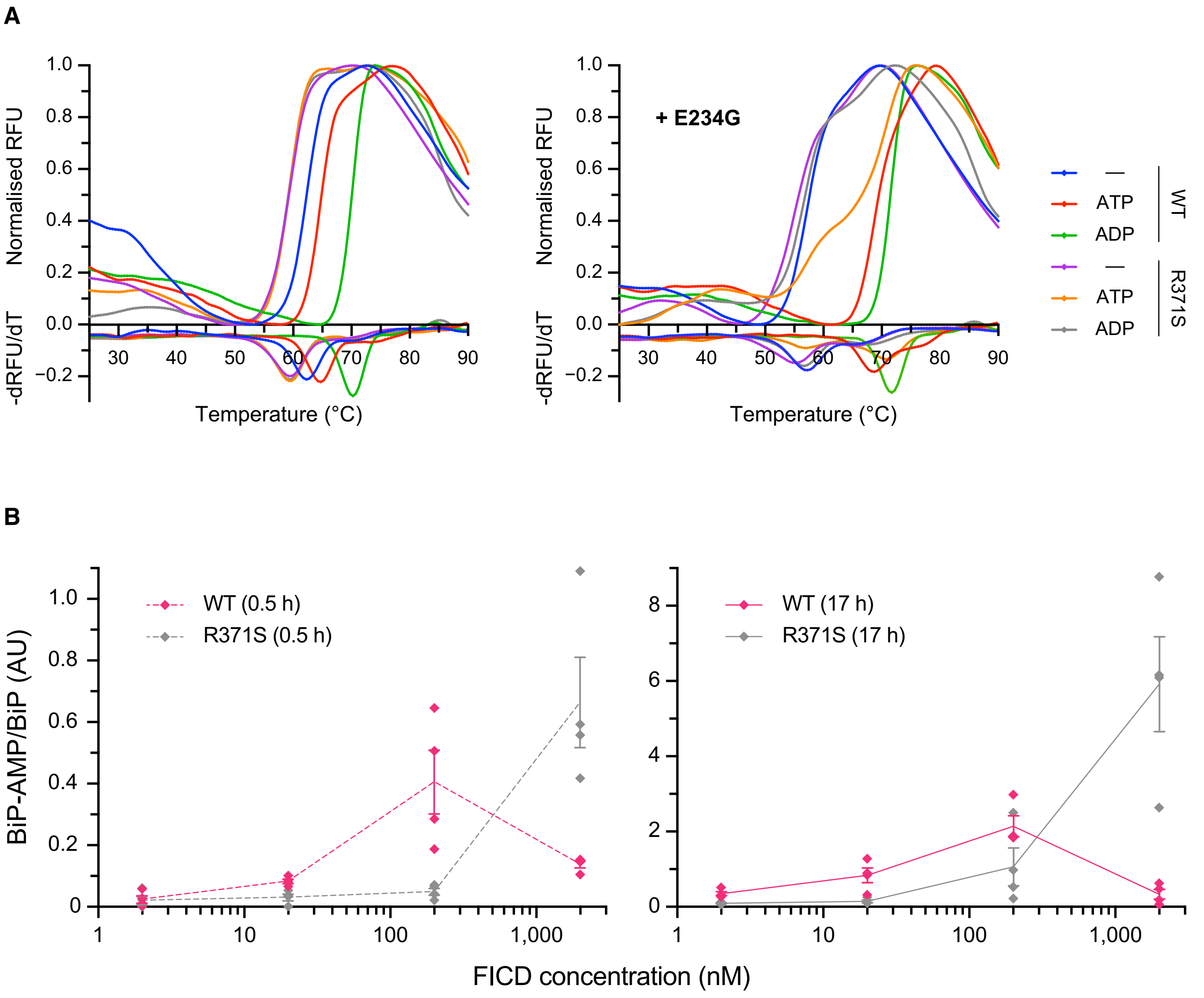

Figure EV2. Effect of the R371S mutation on stability and activity of FICD.

A Representative (of experiments reproduced three time), normalised differential scanning fluorimetry (DSF) melt curves of the indicated FICD proteins (1 μM) in presence and absence of nucleotide (2.5 mM), shown above their corresponding negative first-derivatives. Note, FICD$^{E234G\text{-}R371S}$ displays a non-uniform T_m shift in response to ATP.

B Quantification of the AMPylated BiP signals relative to total BiP of experiments displaying the same data in Fig 3C (mean values ± SEM, n = 4, biological replicates), but split into the two time points of the experiment and presenting the values from all four replicates.

 © 2023 The Authors
