# Supplementary material for: Infancy‐onset diabetes caused by de‐regulated AMPylation of the human endoplasmic reticulum chaperone BiP
Source: EMBO Mol Med. 2023 Jan 27;15(3):e16491. doi: 10.15252/emmm.202216491 (PMC9994480; doi:10.15252/emmm.202216491)
Supplement: Supplementary file 6 [file EMMM-15-e16491-s004.pdf]

EMBO Molecular Medicine

# Infancy-onset diabetes caused by de-regulated AMPylation of the endoplasmic reticulum chaperone BiP

Luke Perera, Andrew Hattersley, Heather Harding, Matthew Wakeling, Sarah Flanagan, Mohsina Ibrahim, Jamal Raza, Alice Gardham, David Ron, and Elisa De Franco
**DOI: 10.15252/emmm.202216491**

*Corresponding authors: David Ron (dr360@medschl.cam.ac.uk) , Elisa De Franco (E.De-Franco@exeter.ac.uk)*

## Review Timeline:

| | |
|---|---|
| Submission Date: | 24th Jun 22 |
| Editorial Decision: | 27th Jun 22 |
| Revision Received: | 31st Oct 22 |
| Editorial Decision: | 28th Nov 22 |
| Revision Received: | 8th Dec 22 |
| Accepted: | 9th Dec 22 |

*Editor: Zeljko Durdevic*

## Transaction Report:

(Note: The initial review process for this manuscript took place with another journal. The initial reviewers' comments and authors' responses for this article have been made available. All referees have been contacted and agreed to have their comments published with this article. With the exception of the correction of typographical or spelling errors that could be a source of ambiguity, letters and reports are not edited. Depending on transfer agreements, referee reports obtained elsewhere may or may not be included in this compilation. Referee reports are anonymous unless the Referee chooses to sign their reports.)

27th Jun 2022

Dear Dr. Ron,

Thank you for submitting your manuscript to EMBO Molecular Medicine. I have now carefully read your manuscript and discussed it with the other members of our editorial team. We appreciate that your study shows the first evidence that links FICD mutation to human disease, but we also agreed that major revisions addressing the reviewers' concerns in full will be necessary for considering the manuscript in our journal.

EMBO Molecular Medicine encourages a single round of revision only and therefore, acceptance or rejection of the manuscript will depend on the completeness of your responses included in the next, final version of the manuscript. For this reason, and to save you from any frustrations in the end, I would strongly advise against returning an incomplete revision.

Further consideration of your manuscript will entail entail a second round of review and will depend on addressing the following points:
- Please provide more mechanistic insight by testing whether the persistence of a pool of AMPylated BiP caused by the R371S mutation compromises cell function using appropriate cell lines.
- Please pay particular attention to appropriate citation of the published literature.
- Please provide additional clarifications and discussion to other referees' points.

Additional experiments that further strengthen the main conclusions of the study are of course appreciated. We would welcome the submission of a revised version within three months for further consideration. Please let us know if you require longer to complete the revision.

Please use this link to login to the manuscript system and submit your revision: https://embomolmed.msubmit.net/cgi-bin/main.plex

I look forward to receiving your revised manuscript.

Yours sincerely,

Zeljko Durdevic

Zeljko Durdevic
Editor
EMBO Molecular Medicine

PBP_V1_2

EMM-2022-16491

30-Oct-2022

# Point-by-point response to reviewer's comments

## Overview:

The reviewers accepted that the link between the FICD p.(Arg371Ser) mutation and the trait exhibited by the study subjects (and its mode of its inheritance) are convincingly established. They also accepted that the defect wrought by the mutation in FICD's enzymatic activity has been addressed by in vitro and cell-based studies. Furthermore, they recognised the significance of these findings as an advance in knowledge.

Beyond a variety of specific issues (addressed in the pages that follow) they expressed two important concerns:

The first relates to individuals heterozygous for the FICD p.(Arg371Ser) mutation. As the parents of the affected homozygous individuals (obligate carriers of the genetic trait) are phenotypically normal, the question arises as to how this might be reconciled with the proposed mechanism of action of the mutant enzyme, which we find to be a change-of-function (as opposed to a simple loss of function). In the original version we argued that this likely reflects an ability of the wildtype copy to reverse the adverse consequences of the mutant copy. Whilst the reviewers accepted the plausibility of this claim, they challenged us to support it experimentally. Drawing on the suggestion of reviewer #2 we have now provided in vitro biochemical evidence that even when present at sub-stoichiometric amounts the wildtype enzyme can reverse the AMPylation bias imposed by the mutant copy. The experimental data is presented in new panel 3F.

The second concern relates to the extent to which a functional defect arising from the FICD p.(Arg371Ser) mutation in vivo (i.e. the phenotype of affected individuals) can be attributed to its biochemical properties demonstrated in vitro. In the original version we argued on the grounds of plausibility: the mutant FICD imposes hyper-AMPylation of BiP. AMPylated BiP is inactive. BiP is an essential chaperone in the endoplasmic reticulum. Hence it stands to reason that unleashing a rogue enzyme [FICD p.(Arg371Ser)] that inactivates BiP will plausibly account for the defect observed. All the more so as the phenotype of this mutation is conspicuously manifest within acell type - the beta cell - that is known to be particularly sensitive to perturbed function of the endoplasmic reticulum. Despite what we regard as a logical and sufficient set of arguments to support the claim made in the title and abstract, the reviewers tasked us with providing further evidence for a link between a BiP hyperAMPylation state imposed by dysregulated FICD and cellular dysfunction. We interpret the implicit justification for this task to be the concern that FICD may have other substrates whose hyperAMPylation might contribute to the phenotype.

To date no stoichiometrically-significant AMPylation of cellular proteins, apart from BiP, has been demonstrated. The evidence for existence of FICD substrates, other than BiP, rests largely on experiments in which deregulated and often mislocalised FICD is brought into contact with cellular proteins under non-physiological conditions. If one merely tracks the signal arising from incorporation of tritiated thymidine (which labels adenosine pools) into non-nuclear proteins (as has been done by Carlson and Lazarides forty years ago, PMID: 6576354), a single protein is detected – BiP (see figure 1 in their paper). When used as a

probe in immunoblots of cell lysates, the MoAb 17G6, which non-specifically recognises AMPylated proteins (PMID 33299971), picks up a single dominant species – BiP (see figure 5 here).

Despite these a priori reservations, we sought to address the reviewers' concern. To do so we exploited recent insights into the mode of BiP binding by FICD, to test whether point mutations that compromise a BiP-contacting surface of FICD reverse the ability of hyperAMPylating FICD to compromise secretion. New figure 6 shows that this is indeed the case. Thus, our conclusion that infancy-onset diabetes observed in individuals homozygous for the FICD p.(Arg371Ser) mutation is caused by de-regulated AMPylation of the human endoplasmic reticulum chaperone BiP, rests not only on a plausible interpretation of the properties of the mutant enzyme in vitro but is also buttressed by evidence that the cellular dysfunction requires specific residues of the mutant enzyme that contact BiP (but otherwise have no effect on the catalytic activity).

The existence of alternative substrates of FICD cannot be firmly refuted. Furthermore, it is formally possible that FICD-mediated hyperAMPylation of other yet-to-be discovered cellular proteins (whose AMPylation also happens to be sensitive to point mutations in FICD that compromise BiP AMPylation) contributes to the phenotype of the individuals described here. But with these caveats in mind, we believe our conclusion to be supported to a standard that licenses their publication in EMM.

## Reviewer 1

In this manuscript, Perera et. al. show that a single point mutation in the FICD protein (R371S) results in misregulation of BiP's AMPylation, which correlates with severe phenotypes such as early-onset diabetes and neurodevelopmental defects. R371S diminishes both the AMPylation and the deAMPylation activities of FICD, with the deAMPylation activity being more severely affected. This results in high levels of AMPylated BiP (compared to WT), in response to stress.

The strength of the paper lies in the beautiful human genetics data, as this is the first evidence that links FICD mutation to human disease. The weakness, however, is that there is very little mechanistic data that links the misregulation of AMPylated Bip to any of the disease phenotypes.

1. My major concern is that the mechanistic link between the R371S mutation and the diseases that have been correlated to it is missing. For e.g., why are slightly high levels of AMPylated BiP (based on their knock-in data Figure 5) causing diabetes? The authors never test insulin-producing beta cells and cells relevant to neurological function to show why this change is significant.

New Figure 6 in the revised version addresses this concern.

An exploration of the role of the mutation in beta cells is potentially of interest, but maintaining physiological relevance of the cells (i.e., stimulus-coupled insulin secretion) whilst genetically manipulating cultured beta cells is far from trivial. The uncertainties in outcome and the investment in time renders this a project for the future.

2. In figure 5b, when they treat the CHO cells with Thapsigargin, is there any difference in CHOP, ATF4, XBP1 between the parental cells and the knock-ins? When they overexpress them in figure 4, there seems to be no difference in CHOP and XBP1 levels, between WT vs R371S expression in these cells. Why then are the beta cells so sensitive to this mutation?

Whilst the mutant cells have a trend towards higher activity of UPR pathways, this is not sufficiently conspicuous a feature to be tracked reliably, hence it is not mentioned in this paper. Diabetes even though arising at a very young age in these individuals, is a progressive defect. Perhaps UPR activity is too insensitive a measure of the relevant cellular dysfunction to register in the cell culture system used here. Notice that we make no claim that conventional ER stress accounts for the development of the phenotype, only that BiP hyperAMPylation is an important contributor.

## Reviewer 2:

This manuscript by Perera et al provides first-of-its-kind evidence linking the function of the human fic-type AMPylase, FICD, to infant-onset clinical conditions. FICD is an enzyme that catalyzes both the transfer and removal of adenosine monophosphate (AMP) to/from threonine side chains of recipient proteins. Earlier studies have connected FICD activity to neurogenesis and cytokine secretion. In this study, the authors describe a FICD(R371D) mutation that is found in five patients suffering from infant-onset diabetes and developmental delay. The authors use a convincing combination of in vitro biochemistry, cell-based, and structural approaches to show that FICD(R371D) is deficient in deAMPylation activity, while retaining limited AMPylase activity. This study, guided by human genetics, provides interesting additional data to the ongoing discussion of the physiological role of

FICD. The rigor of the presented work is high and the conclusions of the paper are mostly supported by the presented data.

In its current form, the paper contains convincing human genetics data and rigorous mechanistic work showing how FICD(R371S) modifies BiP in vitro and in cellulo. What is missing is any attempt to show that the discussed diseases are indeed linked to alteration in BiP AMPylation. One way to do this might be for the authors to use one of the available Beta-cell lines (MIN6, betaHC-1, INIS-1, etc) - all of which retain glucose-stimulated insulin secretion - knock-in the FICD(R371S) mutation and determine if insulin secretion levels are affected. This or related experiments should provide experimental support for the speculative statement shared in the discussion section on page 11: "The disease mechanism likely consists of an intra-organellar perturbation that comprises both insulin-producing beta cells and cells relevant to neurological development and/or function."

On page 11: the authors write: "This scenario is consistent with the recessive nature of the genetic disease. Whilst the imbalance in antagonistic enzymatic activities is intrinsic to the FICDR371S mutant enzyme, the product of a wild-type FICD allele, a powerful deAMPylase (Preissler et al., 2017a), would have no difficulty in buffering the weak AMPylation bias imposed by a single allele of the mutant FICD." This is an interesting and plausible hypothesis. To provide experimental support for this statement, the authors should perform experiments analogous to those shown in Figure 3A using mixtures of FICD:FICD(R371S) in different ratios in the same reaction.

Following this excellent suggestion, we have performed the mixing experiment suggested (new Figure 3F). The observations support the conclusion that the wildtype enzyme can antagonise the hyperAMPylation state otherwise imposed by the mutant version.

Concerns related to data interpretation:

- Figure 3a shows that FICD(R371S) elutes as a dimer at 2,500nM concentration and still remains mainly dimeric at 500nM concentration (similar to FICD wild-type). Figure 3c then shows that particularly at very high concentrations (2,000nM), FICD(R371S) is very effective in AMPylating BiP, even though FICD(ER371S) is expected to be dimeric at this concentration. This finding is in direct conflict with the proposed model that monomeric FICD acts as an AMPylase, while dimeric FICD acts primarily as a deAMPylase.

Monomeric FICD is a better AMPylase and dimeric FICD a better deAMPylase, but both oligomeric states of the enzyme still retain some measure bifunctionality. Therefore, it is not surprising that a mutant enzyme that intrinsically lacks any deAMPylation activity will, over time, promote a pool of AMPylated BiP, even if present at concentrations in which it is principally dimeric.

- Figure 4a: BiP-AMP levels for lanes 3 (FICDE234G) and 4 (FICDR371S) indicate slightly higher BIP-AMP levels in lane 3. Assays in figure 4B, however, show a stark change in how CHOP::GFP and XBP-1::GFP react to the presence of FICD(E234G) and FICD(R371S). A potential explanation for this is that additional proteins might be AMPylated, particularly by FICD(E234G), which then co-trigger the observed changes in CHOP::GFP and XBP-1::GFP signal.

As noted in the overview (above), there is nothing to argue against the formal possibility of a contribution of substrates other than BiP to the phenotype arising from hyperAMPylating alleles of FICD. This point is plainly conceded in discussion of the paper. However, the

reversal of the defect in secretion by compounding point mutations that compromise FICD's ability to contact ATP-state (domain-docked) BiP (new Figure 6), argue that such yet-to-be-discovered substrates would also have to rely on an identical mode of substrate recruitment – an important constraint on this proposition.

Another concern is in how the authors embed their new data in the context of peer-reviewed published literature on protein AMPylation:

- Introduction, page 2, the authors write: "AMPylation, which occurs on a single residue, THR518, locks BiP...". Papers by Sanyal et al. and Ham et al. show that Thr356 is AMPylated, too.

We have modified the text to indicate that here we are concerned with AMPylation of Thr518, thus side-stepping this issue

- On page 2, the authors write: "levels of modified (AMPylated) BiP are regulated...", citing Hendershot et al., 1988 and Laitusis et al., 1999. On page 9, the authors write "as previously noted: Laitusis et al., 1999)", referring to a lower BiP-AMP signal. This is misleading to the unknowing reader. Papers by Hendershot et al., 1988, and Laitusis et al., 1999, do not report on any experiments focusing on BiP AMPylation. Hendershot et al. show that BiP is phosphorylated and labelled with [H3]-Adenosine. Laitusis et al. report ADP-ribosylation of BiP, not AMPylation. While in both cases it is a possibility that the authors indeed observed AMPylation, these papers do not conclude that BiP is AMPylated. As such, these papers do not provide direct support for the statements in which context they are cited.

- Page 12: The authors write "Our focus on BiP is justified by the observation that it is the main/only protein to undergo detectable incorporation of AMP (Carlsson and Lazardies, 1983' and 17G6 immunoblots here)" The "main/only" should be changed to "main" as there is a strong and still growing body of published literature showing that many other proteins are AMPylated. This is not a BiP-specific modification. Further, the "(Carlsson and Lazardies, 1983' and 17G6 immunoblots here)" part seems to be a missed reference to be added. Citing Carlsson and Lazardies 1983 in this context, however, seems again incorrect as Carlsson & Lazardies conclude in their 1983 paper that BiP is ADP-ribosylated, not AMPylated. They even write in their paper: "To verify that SP83 is ADP-ribosylated (and not modified in any other way involving adenosine and 5'-AMP), we..."

The revised version specifically cites experimental work concerning the possible existence of alternative substrates for FICD.

In the past Lazirides, Hendershot, Brostrom (and indeed ourselves, see PMID: 22869598), thought they were looking at ADP ribosylation (or phosphorylation) of BiP. We now know that we were all observing evidence of BiP AMPylation (and on Thr518, at that, see PMID: 9495282) as recently reviewed in PMID: 36041787. The beauty of well-done experiments is that the observations hold, even if the conclusions are proven to be wrong. The seminal work of these researcher is the foundation of our knowledge, and we deem it legitimate to revisit their data and reinterpret it in light of new knowledge.

- Table 1: please add column titles.

Thanks. Done

## Reviewer 3:

This straightforward and generally well-written manuscript from the De Franco and Ron groups describes the identification of a homozygous Arg371Ser mutation in FICD associated with infancy-onset diabetes mellitus and severe neurodevelopmental delay in 5 individuals from consanguineous families. Biochemical characterization of the impact for this point mutation uncovers an elimination of FICD deAMPylation activity, with a limited impact on FICD AMPylation activity. Biochemical data show an ability of the R371S mutant to facilitate AMPylation, and these data are complemented by experiments establishing a disruption in deAMPylation activity. Stress conditions in cells that normally promote deAMPylation show little impact for the R371S mutant, consistent with a compromised deAMPylation function for the R371S mutant.

The authors outline a model postulating the dynamics for BiP AMPylation and the consequences for insulin biogenesis in wild-type versus FICD mutant pancreatic beta cells. Given the prior modest defects reported for a FICD null mouse or flies lacking ficd, these data imply an important distinction between abrogation of AMPylation and de-regulation of AMPylation, and they provide insight and advance our knowledge of the role of FICD activity organism physiology. In turn, the results suggest the potential to bypass defects associated with altered AMPylation activities in cells by abrogating AMPylation activity for therapeutic purposes.

The conclusions of the paper are generally well supported by the data. Some data analysis, and how the data analysis in questions relates to their model, should be clarified.

1. It is not clear how quantification of the images corresponding to the Fig. 5c blot was completed, given an absence of detectable BiP-AMP on the representative western image. Are the RU values in panel 5d reflective of background pixels on the western? Was a detectable signal present in other experiments? Quantification of a longer exposure?

This is a good point. Whilst $FICD^{R371S}$ cells invariably have a pool of AMPylated BiP (which, as we have shown, is unresponsive to stimuli that normally promote its dissipation) under seemingly basal conditions wildtype cells have variable levels of AMPylated BiP detected by the 17G6 MoAb. We imagine this to reflect the fact that subtle differences in cell culture conditions lead to variation in the activity of wild-type FICD. This accounts for the basal signal in the bar diagram, alluded to by the reviewer. We have modified the text describing these observations to clarify this point.

2. When expressed at the endogenous FICD locus, the R371S mutant showed a higher baseline BiP-AMP relative to parental cells as concluded via western blotting for BiP-AMP (Fig 5b-c). However, when quantified in a later experiment and normalized to BiP levels, (see point 1), the relative proportion of AMPylated BiP in cells appears comparable in the parental and mutant strains. Fig 5b suggests also that total BiP levels may be lower in the mutant cells. These latter observations are not discussed in the text. How does one parse the ratio of AMPylated BiP versus the total amount of AMPylation BiP in cells with respect to the proposed model?

Please see response to the comment above.

When normalised to total protein and within the resolution of the assay, we are not convinced that wild-type and FICDR371S cells have consistently different levels of total BiP.

The discussion of the relationship between oligomerization, FICD activity, and the Arg371 mutation initially confused this reviewer (page 7). Starting after the sentence: "Together these findings (findings is missing an 's') point to an important role...". While upon re-review, the intent was clearer, a slight reorganization may make this easier for the naïve reader to follow.

Specifically:

(a) The authors establish an absence of gross differences in monomer-dimer profile for the FICD wild type and mutant proteins (Fig. 3a). Moving directly from these data to the discussion of the predominance of deAMPylation activity at high concentrations of wild-type FICD (dimers), and the loss of activity observed for the mutant at high concentrations of FICD (despite no obvious change to dimerization), might better connect these concepts (versus a discussion of the thermostability sandwiched between the discussions of panels Fig. 3a and 3b). Perhaps beginning with the thermostability data and then moving to the effect of nucleotide followed by the discussion of the dimerization and relationship to the FICD bifunctionality would allow for a clearer initial read.

Thanks. Acting on this suggestion we have modified the order in which the data is presented.

(b) Although not necessary, given there is an open panel (blank space) in the current Fig. 3, an image of the relationship between oligomerization and AMPylation/deAMPylation could also be helpful.

The blank space in Figure 3 now accommodates new panels 3F & 3G. Nonetheless we recognise the challenge of communicating the complexities of FICD biochemistry to a broad readership and hope we have improved this aspect by following the reviewer's suggestion, above.

28th Nov 2022

Dear Dr. Ron,

Thank you for the submission of your revised manuscript to EMBO Molecular Medicine. I am pleased to inform you that we will be able to accept your manuscript pending the following final amendments:

1) As you will see from the reports below, all three referees support publication of the study raising some minor concerns. Please address all the points and implement all referees' suggestions.
2) Manuscript format: Please format your manuscript according to our guidelines outlined here:
https://www.embopress.org/page/journal/17574684/authorguide#researcharticleguide
3) Figures: Please upload individual, high-resolution main and EV Figure files including Figure 7, which is not provided in combined figures .pdf file. Please check "Author Guidelines" for more information:
https://www.embopress.org/page/journal/17574684/authorguide#figureformat
4) In the main manuscript file, please do the following:
- Correct/answer the track changes suggested by our data editors by working from the attached document.
- Add up to 5 keywords.
- Add callouts for Figures 4C and 6A, B and C.
- Correct callout of Suppl. Table 3 to EV Table 3.
- Delete the list of suppl. tables in the manuscript text.
- In M&M, please include statement that in addition to WMA Declaration of Helsinki the experiments also conformed the Department of Health and Human Services Belmont Report.
- Author contributions: Please remove it from the manuscript and specify author contributions in our submission system. CRediT has replaced the traditional author contributions section because it offers a systematic machine-readable author contributions format that allows for more effective research assessment. You are encouraged to use the free text boxes beneath each contributing author's name to add specific details on the author's contribution. More information is available in our guide to authors:
https://www.embopress.org/page/journal/17574684/authorguide#authorshipguidelines
- Raw data from large-scale datasets (genome sequencing) should be deposited in one of the relevant databases and made freely available prior the publication of the manuscript. Use the following format to report the accession number of your data:

The datasets produced in this study are available in the following databases:
[data type]: [full name of the resource] [accession number/identifier] ([doi or URL or identifiers.org/DATABASE:ACCESSION])

Please check "Author Guidelines" for more information.
https://www.embopress.org/page/journal/17574684/authorguide#availabilityofpublishedmaterial
5) Synopsis: Every published paper now includes a 'Synopsis' to further enhance discoverability. Synopses are displayed on the journal webpage and are freely accessible to all readers. They include separate synopsis image and synopsis text.
- Synopsis image: Please provide a striking image or visual abstract as a high-resolution jpeg file 550 px-wide x (250-400)-px high to illustrate your article.
- Synopsis text: Please provide a short standfirst (maximum of 300 characters, including space) as well as 2-5 one sentence bullet points that summarise the paper as a .doc file. Please write the bullet points to summarise the key NEW findings. They should be designed to be complementary to the abstract - i.e. not repeat the same text. We encourage inclusion of key acronyms and quantitative information (maximum of 30 words / bullet point). Please use the passive voice.
- Please check your synopsis text and image before submission with your revised manuscript. Please be aware that in the proof stage minor corrections only are allowed (e.g., typos).
6) For more information: This space should be used to list relevant web links for further consultation by our readers. Could you identify some relevant ones and provide such information as well? Some examples are patient associations, relevant databases, OMIM/proteins/genes links, author's websites, etc...
7) As part of the EMBO Publications transparent editorial process initiative (see our Editorial at http://embomolmed.embopress.org/content/2/9/329), EMBO Molecular Medicine will publish online a Review Process File (RPF) to accompany accepted manuscripts. This file will be published in conjunction with your paper and will include the anonymous referee reports, your point-by-point response and all pertinent correspondence relating to the manuscript. Let us know whether you agree with the publication of the RPF and as here, if you want to remove or not any figures from it prior to publication. Please note that the Authors checklist will be published at the end of the RPF.
8) Please provide a point-by-point letter INCLUDING my comments as well as the reviewer's reports and your detailed responses (as Word file).

I look forward to reading a new revised version of your manuscript as soon as possible.

Yours sincerely,

Zeljko Durdevic

Zeljko Durdevic
Editor
EMBO Molecular Medicine

***** Reviewer's comments *****

Referee #1 (Remarks for Author):

The revised version of this manuscript by Perera and colleagues contains convincing additional experimental data supporting the originally proposed conclusions. All my initial experimental concerns were sufficiently addressed.

There are two minor points regarding citation of relevant literature I'd like to raise, and I'll leave it to the editor to decide whether or not these points are to be addressed:

1) On page 10 in the revised manuscript, the authors cite work from a single lab when referring to the possible existence of additional FICD targets, almost implying that this finding is not replicated by other groups. However, additional FICD targets have been described by several labs and studies, including Truttmann et al., Kielkowski et al., McCaul et al., Sanyal et al., Lewallen et al., Broncell et al., Höpfner et al., (showing that AMPyltion-specific Ab 17G6 recognizes multiple bands in most tested cell lysates), Becker at al., etc. Thus, adding a few more citations would be only fair to the reader.

2) In the rebuttal, Perera and colleagues write: "In the past Lazirides, Hendershot, Brostrom (and indeed ourselves, see PMID: 22869598), thought they were looking at ADP ribosylation (or phosphorylation) of BiP. We now know that we were all observing evidence of BiP AMPylation (and on Thr518, at that, see PMID: 9495282) as recently reviewed in PMID: 36041787. The beauty of well-done experiments is that the observations hold, even if the conclusions are proven to be wrong."
I agree that the de novo interpretation of "old" data is a good strategy and I personally believe that Lazirides, Hendershot, and Brostrom indeed did observe signs of BiP AMPylation in their early work. However, the cited studies in question do not provide indisputable experimental support for BiP AMPylation but equally well support the original interpretation of BiP ADP ribosylation

and/or phosphorylation. As such, I remain firm in my believe that these studies are inappropriately cited in this manuscript by Perera et al and should be removed from the sections in question (see original comments).

Referee #2 (Comments on Novelty/Model System for Author):

Human genetics data reveal a relatively rare, and not previously described, homozygous FICD mutation associated with infancy-onset diabetes mellitus and severe neurodevelopmental delay. Biochemical characterization and in cell studies correlate altered FICD activity to changes in BiP function.

Referee #2 (Remarks for Author):

The current manuscript revised for EMBO Molecular Medicine addresses my concerns raised during the initial review. While I feel the original suggestion(s) that following secretion in a FICD mutant beta-cell line would further strengthen the manuscript, I accept that these experiments may technically be beyond the scope of the current manuscript.

A minor issue related to the new data:

While the relevant information on the FICD mutant alleles used in Fig. 6 information can be gleaned from the figure legend, it would be helpful to the naive reader to include the mutant alleles in the main text. Specifically, Fig 6 shows alleles K124E, H131A referred to only in the main text generically as TRP domain mutants.

Referee #3 (Comments on Novelty/Model System for Author):

This paper assigns a new mutation on FICD (R371S) to early-onset diabetes. The quality of the data is superb, the novelty is there even though another paper (Rebelo et al. 2022) showed that BiP inactivation due to loss of De-AMPylation leads to motor neuron disease.

Referee #3 (Remarks for Author):

The authors have successfully addressed the points raised by me, and I am happy to recommend publication.

Reviewer #1:

1) On page 10 in the revised manuscript, the authors cite work from a single lab when referring to the possible existence of additional FICD targets, almost implying that this finding is not replicated by other groups. However, additional FICD targets have been described by several labs and studies, including Truttmann et al., Kielkowski et al., McCaul et al., Sanyal et al., Lewallen et al., Broncell et al., Höpfner et al., (showing that AMPyltion-specific Ab 17G6 recognizes multiple bands in most tested cell lysates), Becker at al., etc. Thus, adding a few more citations would be only fair to the reader.

In deference to the reviewer, we have added citations to papers by McCaul et al and Höpfner et al.

2) In the rebuttal, Perera and colleagues write: "In the past Lazirides, Hendershot, Brostrom (and indeed ourselves, see PMID: 22869598), thought they were looking at ADP ribosylation (or phosphorylation) of BiP. We now know that we were all observing evidence of BiP AMPylation (and on Thr518, at that, see PMID: 9495282) as recently reviewed in PMID: 36041787. The beauty of well-done experiments is that the observations hold, even if the conclusions are proven to be wrong."
I agree that the de novo interpretation of "old" data is a good strategy and I personally believe that Lazirides, Hendershot, and Brostrom indeed did observe signs of BiP AMPylation in their early work. However, the cited studies in question do not provide indisputable experimental support for BiP AMPylation but equally well support the original interpretation of BiP ADP ribosylation and/or phosphorylation. As such, I remain firm in my believe that these studies are inappropriately cited in this manuscript by Perera et al and should be removed from the sections in question (see original comments).

In the revised manuscript, we have clarified that in citing these papers we are referring specifically to data items in them, not to their authors' stated

conclusions. We believe this should assuage even those who share the reviewer's firm views on this matter.

Revewer #2:

While the relevant information on the FICD mutant alleles used in Fig. 6 information can be gleaned from the figure legend, it would be helpful to the naive reader to include the mutant alleles in the main text. Specifically, Fig 6 shows alleles K124E, H131A referred to only in the main text generically as TRP domain mutants.

We have modified the results section to make specific mention of these two alleles.

We are pleased to inform you that your manuscript is accepted for publication and is now being sent to our publisher to be included in the next available issue of EMBO Molecular Medicine.

## EMBO Press Author Checklist

| | |
|---|---|
| Corresponding Author Name: David Ron | |
| Journal Submitted to: EMBO Molecular Medicine | |
| Manuscript Number: EMM-2022-16491 | |

**USEFUL LINKS FOR COMPLETING THIS FORM**
The EMBO Journal - Author Guidelines
EMBO Reports - Author Guidelines
Molecular Systems Biology - Author Guidelines
EMBO Molecular Medicine - Author Guidelines

**Reporting Checklist for Life Science Articles (updated January 2022)**
This checklist is adapted from Materials Design Analysis Reporting (MDAR) Checklist for Authors. MDAR establishes a minimum set of requirements in transparent reporting in the life sciences (see Statement of Task: 10.31222/osf.io/9sm4x). Please follow the journal's guidelines in preparing your manuscript.
**Please note that a copy of this checklist will be published alongside your article.**

**Abridged guidelines for figures**
**1. Data**
The data shown in figures should satisfy the following conditions:
- the data were obtained and processed according to the field's best practice and are presented to reflect the results of the experiments in an accurate and unbiased manner.
- ideally, figure panels should include only measurements that are directly comparable to each other and obtained with the same assay.
- plots include clearly labeled error bars for independent experiments and sample sizes. Unless justified, error bars should not be shown for technical replicates.
- if n<5, the individual data points from each experiment should be plotted. Any statistical test employed should be justified.
- Source Data should be included to report the data underlying figures according to the guidelines set out in the authorship guidelines on Data Presentation.

**2. Captions**
Each figure caption should contain the following information, for each panel where they are relevant:
- a specification of the experimental system investigated (eg cell line, species name).
- the assay(s) and method(s) used to carry out the reported observations and measurements.
- an explicit mention of the biological and chemical entity(ies) that are being measured.
- an explicit mention of the biological and chemical entity(ies) that are altered/varied/perturbed in a controlled manner.
- the exact sample size (n) for each experimental group/condition, given as a number, not a range;
- a description of the sample collection allowing the reader to understand whether the samples represent technical or biological replicates (including how many animals, litters, cultures, etc.).
- a statement of how many times the experiment shown was independently replicated in the laboratory.
- definitions of statistical methods and measures:
  - common tests, such as t-test (please specify whether paired vs. unpaired), simple χ2 tests, Wilcoxon and Mann-Whitney tests, can be unambiguously identified by name only, but more complex techniques should be described in the methods section;
  - are tests one-sided or two-sided?
  - are there adjustments for multiple comparisons?
  - exact statistical test results, e.g., P values = x but not P values < x;
  - definition of 'center values' as median or average;
  - definition of error bars as s.d. or s.e.m.

**Please complete ALL of the questions below.**
**Select "Not Applicable" only when the requested information is not relevant for your study.**

**Materials**

| Newly Created Materials | Information included in the manuscript? | In which section is the information available? (Reagents and Tools Table, Materials and Methods, Figures, Data Availability Section) |
|---|---|---|
| New materials and reagents need to be available; do any restrictions apply? | Yes | All materials are freely available, see Materials and Methods and Table EV2 with plasmids used in the studies |

| Antibodies | Information included in the manuscript? | In which section is the information available? (Reagents and Tools Table, Materials and Methods, Figures, Data Availability Section) |
|---|---|---|
| For **antibodies** provide the following information:<br>- Commercial antibodies: RRID (if possible) or supplier name, catalogue number and or/clone number<br>- Non-commercial: RRID or citation | Yes | See materials and methods and Table EV3 |

| DNA and RNA sequences | Information included in the manuscript? | In which section is the information available? (Reagents and Tools Table, Materials and Methods, Figures, Data Availability Section) |
|---|---|---|
| **Short novel DNA or RNA including primers, probes**: provide the sequences. | Yes | All materials are freely available, see Materials and Methods and described in table EV2 (plasmids) and EV3 (DNA primers) |

| Cell materials | Information included in the manuscript? | In which section is the information available? (Reagents and Tools Table, Materials and Methods, Figures, Data Availability Section) |
|---|---|---|
| **Cell lines:** Provide species information, strain. Provide accession number in repository **OR** supplier name, catalog number, clone number, and/**OR** RRID. | Yes | Table EV3 |
| **Primary cultures:** Provide species, strain, sex of origin, genetic modification status. | Not Applicable | |
| Report if the cell lines were recently **authenticated** (e.g., by STR profiling) and tested for mycoplasma contamination. | Yes | Genomic sequencing validates the species of origin |

| Experimental animals | Information included in the manuscript? | In which section is the information available? (Reagents and Tools Table, Materials and Methods, Figures, Data Availability Section) |
|---|---|---|
| **Laboratory animals or Model organisms:** Provide species, strain, sex, age, genetic modification status. Provide accession number in repository **OR** supplier name, catalog number, clone number, **OR** RRID. | Not Applicable | |
| **Animal observed in or captured from the field:** Provide species, sex, and age where possible. | Not Applicable | |
| Please detail **housing and husbandry conditions**. | Not Applicable | |

| Plants and microbes | Information included in the manuscript? | In which section is the information available? (Reagents and Tools Table, Materials and Methods, Figures, Data Availability Section) |
|---|---|---|
| **Plants:** provide species and strain, ecotype and cultivar where relevant, unique accession number if available, and source (including location for collected wild specimens). | Not Applicable | |
| **Microbes:** provide species and strain, unique accession number if available, and source. | Not Applicable | |

| Human research participants | Information included in the manuscript? | In which section is the information available? (Reagents and Tools Table, Materials and Methods, Figures, Data Availability Section) |
|---|---|---|
| If collected and within the bounds of privacy constraints report on age, sex and gender or ethnicity for all study participants. | Yes | Materials and methods |

| Core facilities | Information included in the manuscript? | In which section is the information available? (Reagents and Tools Table, Materials and Methods, Figures, Data Availability Section) |
|---|---|---|
| If your work benefited from core facilities, was their service mentioned in the acknowledgments section? | Yes | In acknowledgement section |

**Design**

| Study protocol | Information included in the manuscript? | In which section is the information available? (Reagents and Tools Table, Materials and Methods, Figures, Data Availability Section) |
|---|---|---|
| If study protocol has been **pre-registered, provide DOI in the manuscript**. For clinical trials, provide the trial registration number **OR** cite DOI. | Not Applicable | |
| Report the **clinical trial registration number** (at ClinicalTrials.gov or equivalent), where applicable. | Not Applicable | |

| Laboratory protocol | Information included in the manuscript? | In which section is the information available? (Reagents and Tools Table, Materials and Methods, Figures, Data Availability Section) |
|---|---|---|
| Provide DOI OR other citation details if **external detailed step-by-step protocols** are available. | Not Applicable | |

| Experimental study design and statistics | Information included in the manuscript? | In which section is the information available? (Reagents and Tools Table, Materials and Methods, Figures, Data Availability Section) |
|---|---|---|
| Include a statement about **sample size** estimate even if no statistical methods were used. | Yes | In the legend to figures and tables |
| Were any steps taken to minimize the effects of subjective bias when allocating animals/samples to treatment (e.g. **randomization procedure**)? If yes, have they been described? | Not Applicable | |
| Include a statement about **blinding** even if no blinding was done. | Not Applicable | |
| Describe **inclusion/exclusion criteria** if samples or animals were excluded from the analysis. Were the criteria pre-established?<br><br>If sample or data points were omitted from analysis, report if this was due to attrition or intentional exclusion and provide justification. | Not Applicable | |
| For every figure, are **statistical tests** justified as appropriate? Do the data meet the assumptions of the tests (e.g., normal distribution)? Describe any methods used to assess it. Is there an estimate of variation within each group of data? Is the variance similar between the groups that are being statistically compared? | Yes | Figure legends |

| Sample definition and in-laboratory replication | Information included in the manuscript? | In which section is the information available? (Reagents and Tools Table, Materials and Methods, Figures, Data Availability Section) |
|---|---|---|
| In the figure legends: state number of times the experiment was **replicated** in laboratory. | Yes | done |
| In the figure legends: define whether data describe **technical or biological replicates**. | Yes | done |

## Ethics

| Ethics | Information included in the manuscript? | In which section is the information available? (Reagents and Tools Table, Materials and Methods, Figures, Data Availability Section) |
|---|---|---|
| Studies involving **human participants**: State details of **authority granting ethics approval** (IRB or equivalent committee(s), provide reference number for approval. | Yes | Reporting checklist for genetic association study. Based on the STREGA guidelines, deposited with MedRxiv version of this paper |
| Studies involving **human participants**: Include a statement confirming that **informed consent** was obtained from all subjects and that the experiments conformed to the principles set out in the WMA Declaration of Helsinki and the Department of Health and Human Services Belmont Report. | Yes | Methods section |
| Studies involving **human participants:** For publication of **patient photos**, include a statement confirming that consent to publish was obtained. | Not Applicable | |
| Studies involving experimental **animals**: State details of **authority granting ethics approval** (IRB or equivalent committee(s), provide reference number for approval. Include a statement of compliance with ethical regulations. | Not Applicable | |
| Studies involving **specimen and field samples:** State if relevant **permits** obtained, provide details of authority approving study; if none were required, explain why. | Not Applicable | |

| Dual Use Research of Concern (DURC) | Information included in the manuscript? | In which section is the information available? (Reagents and Tools Table, Materials and Methods, Figures, Data Availability Section) |
|---|---|---|
| Could your study fall under dual use research restrictions? Please check biosecurity documents and list of **select agents and toxins** (CDC): https://www.selectagents.gov/sat/list.htm | Not Applicable | |
| If you used a select agent, is the security level of the lab appropriate and reported in the manuscript? | Not Applicable | |
| If a study is subject to dual use research of concern regulations, is the name of the **authority granting approval and reference number** for the regulatory approval provided in the manuscript? | Not Applicable | |

## Reporting

The MDAR framework recommends adoption of discipline-specific guidelines, established and endorsed through community initiatives. Journals have their own policy about requiring specific guidelines and recommendations to complement MDAR.

| Adherence to community standards | Information included in the manuscript? | In which section is the information available? (Reagents and Tools Table, Materials and Methods, Figures, Data Availability Section) |
|---|---|---|
| State if relevant guidelines or checklists (e.g., **ICMJE, MIBBI, ARRIVE, PRISMA**) have been followed or provided. | Not Applicable | |
| For **tumor marker prognostic studies,** we recommend that you follow the **REMARK** reporting guidelines (see link list at top right). See author guidelines, under 'Reporting Guidelines'. Please confirm you have followed these guidelines. | Not Applicable | |
| For **phase II and III randomized controlled trials**, please refer to the **CONSORT** flow diagram (see link list at top right) and submit the CONSORT checklist (see link list at top right) with your submission. See author guidelines, under 'Reporting Guidelines'. Please confirm you have submitted this list. | Not Applicable | |

## Data Availability

| Data availability | Information included in the manuscript? | In which section is the information available? (Reagents and Tools Table, Materials and Methods, Figures, Data Availability Section) |
|---|---|---|
| Have **primary datasets** been deposited according to the journal's guidelines (see 'Data Deposition' section) and the respective accession numbers provided in the Data Availability Section? | Not Applicable | |
| Were **human clinical and genomic datasets** deposited in a public access-controlled repository in accordance to ethical obligations to the patients and to the applicable consent agreement? | Not Applicable | |
| Are **computational models** that are central and integral to a study available without restrictions in a machine-readable form? Were the relevant accession numbers or links provided? | Not Applicable | |
| If publicly available data were reused, provide the respective **data citations in the reference list**. | Not Applicable | |
